# Coping with identity threat and health literacy on the quality of life and mental health in students: Structural equation modeling

**DOI:** 10.1002/npr2.12328

**Published:** 2023-03-14

**Authors:** Mina Ataei, Sara Esmaelzadeh Saeieh, Mansooreh Yazdkhasti, Alireza Jashni Motlagh

**Affiliations:** ^1^ Alborz University of Medical Science Karaj Iran; ^2^ Social Determinants of Health Research Center Alborz University of Medical Sciences Karaj Iran; ^3^ Department of Pediatrics, School of Medicine Alborz University of Medical Sciences Karaj Iran

**Keywords:** health literacy, identity threat, mental health, quality of life

## Abstract

**Aim:**

Adolescents face many challenges when entering university. The aim of this study was determined predictor role of coping with identity threat and health literacy on the quality of life and mental health of students.

**Methods:**

This is a descriptive‐analytical study of the structural equation study. Random sampling was performed on 300 students of Alborz University of medical science who were eligible to enter the study. The link of the questionnaires was provided to the students through social networks. The questionnaires were used to study identity threat, mental health, health literacy, and quality of life.

**Results:**

The results of structural equations showed that health literacy has a negative and significant effect (*β* = −0.22) and identity threat strategy has a negative and significant effect on students' mental health disorder (*β* = −0.53). Mental health disorders have a negative and significant effect on the quality of life in students (*β* = −0.49), and health literacy has a positive and significant effect on the quality of life (*β* = 0.35). Based on Sobel criterion, mental health disorder was a partial mediator for the indirect effect of coping threat strategy on quality of life.

**Conclusions:**

Considering the effect of mental health and health literacy on the quality of life and the effectiveness of coping strategies, it is recommended to evaluate the mental health and health literacy of students in universities and provide educational programs for identity coping in the early years after entering the university.

## Abstract 2

Results of study showed effect of indirect identity threat coping strategy and direct effect of mental health and health literacy on students' quality of life. We suggest to evaluate the mental health and health literacy of students and also provide educational programs for identity coping when entering university.

## Graphical abstract

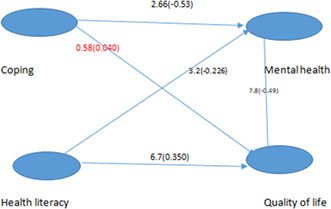

## 1 INTRODUCTION

Quality of life is defined as the individual's understandings of his/her position in life and the culture of the society in which they live. Quality of life is a multidimensional concept and can be measured through physical, physiological, social, and psychological dimensions.^1^ The quality of life is affected by various factors such as stress, anxiety, and mental health disorders.^2^

It is very important to pay attention to the quality of life of students during their studies, which is a very stressful period.^3^ Emotional experiences in the academic environment are related to important results and affect students' health, performance, and progress.^4^ The departure of students from the family environment and the establishment of new friendships in universities, and membership in new social groups, form the future communication of students in universities. When leaving the family, students face pressure from their peers, which causes challenges in the mental health of people. Students enter universities from different cultural background. They leave the family environment and have the first social encounter with others. These environmental factors are a threat to and cause disturbance in the individual's identity, creating sufficient levels of self‐confidence, and self‐efficacy which lead to the correct formation of a person's identity.^5^

In facing identity challenges, people adopt a range of coping strategies, and instead of random and habitual adaptation strategies, it is better to use correct strategies methods and coping styles. Coping is an important variable in relation to the consequences of physical and mental health.^6^ People who effectively cope to their changes experience less anxiety, psychological distress, and depression than others.^7^ Also, these people are less inclined to participating in high‐risk behaviors for health, including alcohol and smoking, high‐risk sexual behaviors, internet addiction, and self‐harming behaviors, and experience a better quality of life.^8^ Various theories and many tools have been proposed to examine coping strategies, all of which are related to health and behavioral outcomes.^9^ The identity process theory is a combinational model of identity construction, threat, and coping in the face of change. Identity threat is a habitual experience in an individual that repeatedly creates trust‐related challenges and creates self‐confidence, self‐efficacy, and a sense of uniqueness.^5^ Students face with a wide range of stress‐related disorders. The university period takes a lot of time and money from the students without guaranteeing satisfaction in the future.^10^ Mental and emotional pressure leads to the deterioration of people's quality of life in various aspects of health, physical, physiological, and environmental.^3^

Health literacy is the most important determinant of life expectancy and also affects the quality of life. Health literacy is the connecting factor between the use of literacy with knowledge, motivation, and ability of a person to access, understand, criticize, and use health information in making decisions in worrying conditions, preventing disease, and improving the quality of life.^11^

Many studies have shown the relationship between health literacy and quality of life, but mixed results have also been reported. Many studies have shown its positive relationship with health literacy,^12^, ^13^, ^14^ while others have shown a negative relationship between quality of life and health literacy.^15^, ^16^ Also, health literacy is an important factor in believing and identifying mental health disorders in order to diagnose manage and prevent mental health problems.^17^

Entering the university, young people face many changes in their lives. For many, entering the university coincides with the transition from adolescence to youth. During this period, participation in new social groups and the development of gender identity occur.^18^ Also, they may face social and psychological factors including misperception of danger, peer pressure, mental health decline, alcohol and drug use, which increase the risk of poor physical and sexual health. Epidemiological data show that students and teenagers face more health and sexual inequalities compared to the general public.^19^ Therefore, based on the importance of social and individual transformations in the age group of students, the present study was conducted aiming at investigating the effect of coping strategies with identity threat and health literacy with the mediation of mental health disorders on the quality of life of students based on structural equations in order to investigate the hypotheses of the study:

Hypothesis 1 Health literacy has an effect on students' mental health.

Hypothesis 2 Coping strategies have an effect on students' mental health.

Hypothesis 3 Mental health affects the quality of life of students.

Hypothesis 4 Coping strategy has an effect on students' quality of life.

Hypothesis 5 Health literacy has an effect on the quality of life of students.

## 2 METHOD

This was a descriptive‐analytical study of the structural equation type and was conducted after obtaining written permission from the ethics committee with the code (IR.ABZUMS.REC.1400.088) on 300 students of Alborz University of Medical Sciences. The sampling method was stratified in the first stage and random in the second. So, based on the total number of students, a certain proportion of the sample size was allocated to each class. In this way, each faculty was considered as a class and the sample was selected based on the number of eligible students. Then, by referring to the education administrator of each faculty, the list of eligible students was selected randomly, and using the table of random numbers and the proportion of students determined in each faculty, the number of samples was selected in the second stage. Sampling was performed after inviting the students and explaining the objectives of the study as well as the confidentiality strategies and obtaining informed electronic consent to participate in the study. The study sample included students of the faculties of medicine, health, and nursing, and midwifery in Alborz University of Medical Sciences, and the criteria for entering the study included studying at the university, first and second year student between 18 and 25 of age.

Exclusion criteria were students with mental problems as confirmed by the physician, guest students from other universities.

The sample size in structural equation modeling was estimated according to Munro's study^20^ and since the total number of items in the questionnaire was 100, three people were sampled for each item (except for item related to demographic information) and taking into account 10% sample loss, the sample size included 310 individuals. The link of the questionnaires was provided to the students electronically through social networks.

### 2.1 Data collection

Data were collected using the following questionnaires:

### 2.2 World Health Organization Quality of Life Questionnaire (WHO‐QOLF)

To examine the quality of life variable, the World Health Organization standard quality of life questionnaire was used. The measurement scale was set based on a five‐point Likert scale from 5 = completely agree to 1 = completely disagree, which consists of 26 items. This questionnaire measures the four dimensions of physical, psychological, social, and environmental health. The score of each dimension is from 4 to 20. This questionnaire was psychometrically evaluated in Iran by Nejat et al. Intracluster correlation and Cronbach's alpha have been reported to be greater than 0.7 in all areas except social relations (0.55).^21^

### 2.3 Coping with Identity Threat Scale (CITS)

To measure compliance with identity threat, the 20‐item identity threat questionnaire of Jaspal and Lopez^5^ was used. This questionnaire is measured based on a five‐point Likert scale from “completely incorrect” to “completely correct.” In the present study, the total score of the questionnaire was examined. Since this questionnaire was used for the first time in Iran, after determining the content validity, the construct validity was analyzed. The confirmatory factor explained in the external measurement model section was confirmed in the results section and the internal reliability was confirmed with Cronbach's alpha coefficient of 0.8.

### 2.4 Heath literacy rate for Iranian adults

This questionnaire is used to measure the health literacy of adults and has 33 items, access (6 items), reading skill (4 items), perceived (7 items), evaluation (4 items), and decision‐making and use of health information (12 items). Its reliability was determined by Montazeri et al. in 2014.^22^

### 2.5 Mental health DASS‐21

The 21‐item DASS‐21 questionnaire was used to measure mental health disorders. It measures stress, anxiety, and depression on a four‐point Likert scale, and the answers range from “not at all, a little, much, to a lot.” Asghari Moghadam et al.^23^ reported the Cronbach's alpha coefficients of depression, anxiety, and stress scales as 0.92, 0.93, and 0.90, respectively, and the re‐test coefficients (with a 3‐week interval) of depression, anxiety, and stress scales as 0.84, 0.89, and 0.90, respectively.

### 2.6 Data analysis

First, the data collected was entered into SPSS‐21. The normality of the data was determined by skewness and kurtosis and the missing data were specified to be replaced with the median and screened based on the data preprocessing indicators. Based on the conceptual model, confirmatory factor analysis was performed first using the external measurement model, and after evaluating the validity and reliability of the model, the relationships between the variables were examined using the internal measurement model. Smart PLS3 software was used to determine the measurement model and structural equations.

## 3 RESULTS

In this study, 56.6% were female students, 53.4% were studying in undergraduate level, and the average age of the participants was 19.11. The demographic characteristics of the students participating in the study are listed in Table 1.

**TABLE 1:** Demographic characteristics.

Variable		F (%)			Mean ± SD
Education	Bachelor	160 (53.4)	Quality of life domain	Total score physical psycho relationship environment	94.3 ± 7.2
	GP	140 (46.6)			26.1 ± 5.2
Residence of students	Dormitory	54 (18)			21.5 ± 4.7
	Personal	14 (4.6)			9.9 ± 2.6
	Family	228 (76)			26.2 ± 4.6
Gender	Female	170 (56.6)	Coping strategy with threat	Total score	71.3 ± 11.2
	Male	130 (43.4)			
Mean ± SD Health literacy	Total score reading access perceived evaluation behavior	141.3 ± 16.6	Mental health score	Total score depression anxiety stress	37.7 ± 14.7
		17 ± 3.07			12.2 ± 5.6
		22.3 ± 4.7			11.1 ± 4.6
		31.6 ± 3.6			14.3 ± 5.5
		13.9 ± 3.1			
		47.2 ± 8.9			

### 3.1 External measurement

The first test for external measurement of the model is the homogeneity test, which is the confirmatory factor analysis. The items that had a factor load of less than 0.7 were removed from the model.^24^ The process of confirmatory factor analysis showed that items 4, 13, 21, and 25 on quality of life, 1 and 14 on health literacy, and 2, 4, 5, 6, 7, 8, 9, 10, 11, 13, 16, 20 on coping strategy are smaller than the cut point of 0.7 and are heterogeneous with questions and should be modified and removed from the model.

For model reliability, Cronbach's alpha tests, composite reliability, and Spearman's RHO‐A correlation coefficient were used. In all cases, the values were greater than 0.7.^25^

In order to determine the convergent validity of the model, the average variance extracted (AVE) test was used.^26^ The test results showed that the values were more than 0.5 and the composite reliability (CR) in all variables was higher than the AVE, so the model had appropriate convergent validity.

### 3.2 Divergent validity

In order to determine the divergent validity, cross‐loading, Fornell Larcker and Hetro trait mono trait (HTMT) tests were used. In the cross‐sectional loading table, each item had the highest factor loading for the variable in question, and the results showed that the questions of each variable diverged or differentiated from the questions of other variables.^27^ Fornell and Larcker's table (Table 2) shows that the square root of the AVE of all variables is higher than the correlation of that variable with other variables. The results of the HTMT test also showed that the correlation between the two variables is less than 0.9 and the divergent validity of the model was also confirmed.

**TABLE 2:** Fornell‐Larcker criterion, CV COM, and SRMR index of model.

	Quality of life	Health literacy	Coping strategy	Mental health disorder	cv‐com	SRMR
Quality of life	0.68				0.38	0.07
Health literacy	0.60	0.60			0.30	
Coping strategy	0.52	0.48	0.61		0.18	
Mental health disorder	−0.68	−0.48	0.64	0.71	0.42	

The crossed validated communality (CV COM) index was used to determine the quality of the external measurement model in terms of predictive quality, which is compared with 0.02, 0.15, and 0.35 according to Chin's standard^28^ and shows weak, medium, and strong quality, respectively. CV COM of all variables was greater than 0.15 (Table 3), so the measurement model had moderate prediction quality. The model fit index was obtained from SRMR whose value should be below 0.08 (SRMR = 0.07; Table 2).^29^

**TABLE 3:** Path coefficient and determined variance of structural equation model.

Hypotheses	*β*	*T* value	*p* value	Result
Health literacy → mental health	−0.22	3.2	0.004	Accepted
Coping strategy → mental health	−0.53	2.66	0.001	Accepted
Mental health → quality of life	−0.49	7.8	0.55	Rejected
Coping strategy → quality of life	0.04	0.58	0.001	Accepted
Health literacy → quality of life	0.35	6.7	0.007	Accepted

Explained variance	*R*‐squared	Adjusted *R*‐squared
Quality of life	0.57	0.56
Mental health	0.44	0.44

### 3.3 Internal structural model

**FIGURE 1:**
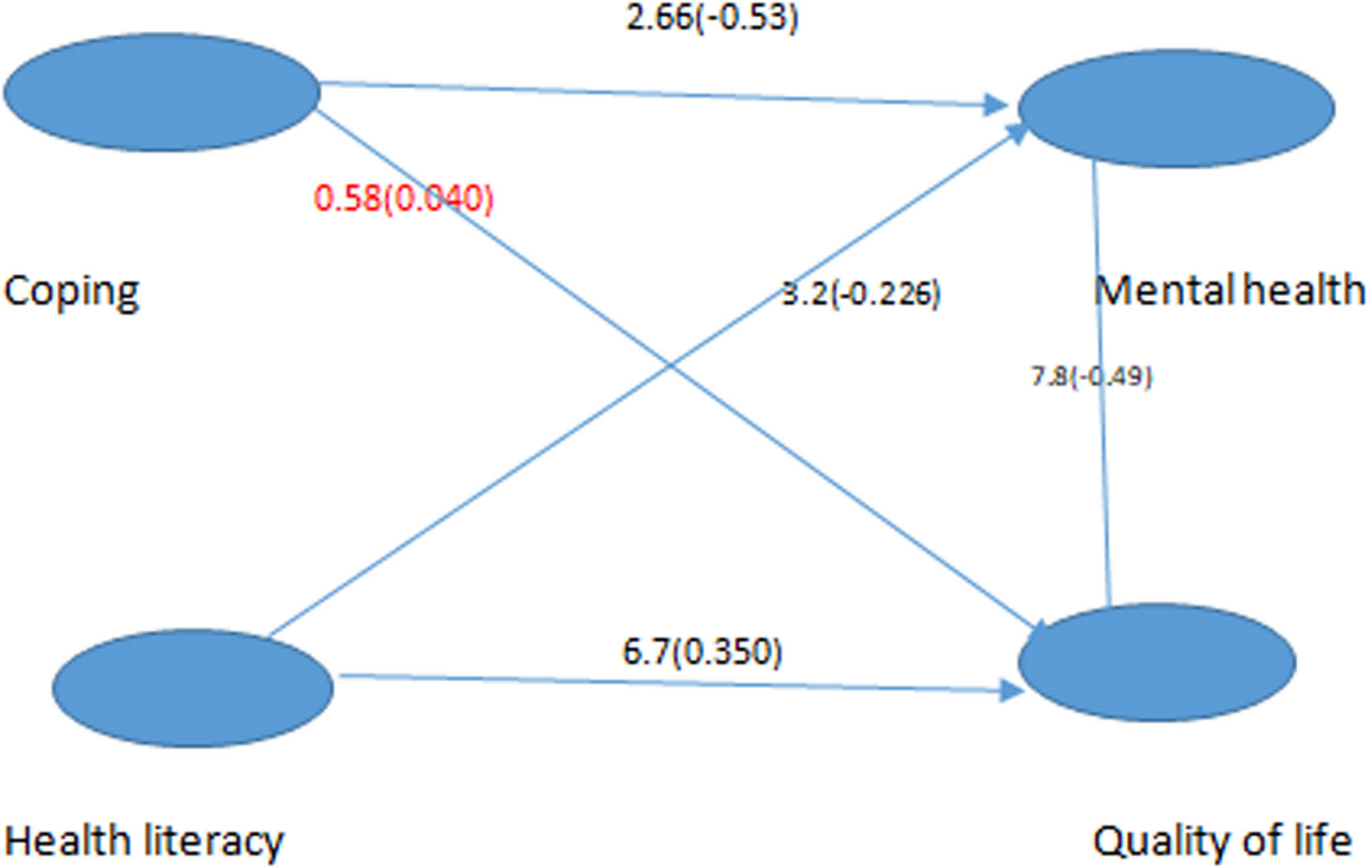
Path coefficient and significance of model.

To determine the relationship between the variables of coping strategies with identity threat, mental health, health literacy, and quality of life, structural model evaluation was used. Results of study showed coping strategy did not have direct effect in student's quality of life. Health literacy has a negative and significant effect on mental health disorder in students (*β* = −0.22), identity threat strategy has a negative and significant effect on students' mental health disorder (*β* = −0.53). Mental health disorders have a negative and significant effect on the quality of life in students (*β* = −0.49), and health literacy has a positive and significant effect on the quality of life (*β* = 0.35). Figure 1 and Table 3 show the significant coefficients and path coefficients of the research variables.

Based on the value of *R* square, the three variables of health literacy, coping strategy with identity threat, and mental health disorder together predict the quality of life of students by 57%. Also, the two variables of health literacy and coping strategy together predict 44% of mental health disorder (Table 3).

### 3.4 Mediating variable analysis

Sobel criterion was used for mediation analysis.^30^ The model was run once without the presence of mental health disorder, and both direct paths of health literacy on quality of life and coping strategies on quality of life were significant according to the *t*‐value. With the mental health disorder in the model as the mediator, the coping threat strategy path on the quality of life was significant, and for this reason, the Variance Accounted for Index was calculated (VAF = 0.59). Also, the Sobel criterion, which was between 0.2 and 0.8, indicated a partial mediation. For the indirect path, health literacy did not mediate quality of life.

## 4 DISCUSSION

The results of the present study showed that coping strategy with identity threat has a negative and significant effect on mental health disorders. The results of a study in 2017 showed that lack of self‐love and emotional maladaptation are the most significant predictors of depression, stress, and anxiety in university students, and the coping strategy was used as the strongest mediator in predicting mental health disorders.^31^

In a study, it was shown that training coping skills reduces the symptoms of mental disorders and increases the mental health of students.^32^ It was also shown in a study that the increase of coping strategies causes an increase in awareness, a positive attitude toward understanding changes and choosing a suitable strategy for the early prevention of mental health problems.^33^

The events and conditions of life can create challenges in the sense of identity and create thoughts about how to understand one‐self and be understood by others.^34^ Due to coping strategies, people will be able to deal with unavoidable problems with others at the social level and should also adapt to themselves, so that these problems do not cause them to be separated from society.^18^

The results of the present study showed that mental health disorder has a negative and significant effect on the quality of life. The results of a study in the Philippines in 2020 showed that the quality of life is significantly and positively related to mental health disorders, anxiety, depression, loss of behavioral control, and life satisfaction. The satisfactory feelings of healthiness and stress have significant positive and negative relationships with the quality of life, respectively, among the youth.^35^

The results of the present study showed that health literacy has a positive and significant effect on the quality of life.

The results of a study in 2022 showed that there is a significant relationship between the level of health literacy and the dimensions of quality of life (physical dimension, psychological dimension, and overall quality of life), so that people with higher health literacy have higher physical and mental quality of life as well as higher health‐related quality of life.^36^ It has been shown in a study that people with sufficient health literacy had a higher quality of life, and there is a relationship between health literacy and the physical and mental aspects of quality of life.^37^

It was shown in a systematic review that health literacy was related to quality of life, and health knowledge, health behavior, health beliefs, and health skills were related to quality of life. There is, however, a need for more studies.^2^ In a study, Song^38^ showed that the relationship between health literacy and quality of life is only 0.07, but it was shown in a study that this relationship was stronger in skills and quality of life. In addition, in health literacy, health skills improve health status and the quality of life.^39^

The results of the present study showed that health literacy has a negative and significant effect on mental health disorders. The results of a study in 2019 showed that 41% of adolescents' mental health is justified by health literacy, which has an explanatory role in improving mental health, and health literacy and knowledge about acquiring and maintaining mental health is an essential component in the field of mental health services.^35^ Considering the effect of mental health, people with health literacy are less affected by feelings of malaise, emotional exhaustion, and psychosomatic complaints.^40^

In this study, coping strategies were not directly related to the quality of life. They affect the quality of life through an indirect path by influencing mental health disorders. Based on the mediation analysis, it was a partial mediator. In a study, it was shown that the difference between the intensity of stress and coping strategies affects the quality of life of students. The reason for the lack of relation can be the lack of measurement of identity threatening cases and lack of receiving sufficient training in the field of coping strategies.^41^ During the last years of adolescence, teenagers spend most of their time in the university and interacting with peers, so the ability of social interaction upon entering university affects people's mental health.^42^ Coping strategies include those that affect people's performance at different levels (cognitive and affective) of intrapersonal, interpersonal, and intragroup relations. Also, the other four levels of coping strategies include self‐awareness about denial, secretiveness, self‐reconstruction, rethinking, and change, which are is related to identity threat and psychological distress.^5^ Therefore, focusing on identity threat cases and their coping and training strategies affects the mental health and identity threat of students and, indirectly, affects the quality of life of students.

The strange of study was assess three variables predicting role by equation structural model in description of student's quality of life.

One of the limitations of this study was that the variable of identity threatening factors has not been investigated, so it is suggested that identity threatening factors be investigated in future studies as well. The second limitation of this conceptual model is the three variables of health literacy, coping strategy, and mental health disorder, predict a total of 57% of the quality of life of students, so it is suggested to conduct further studies to identify other variables affecting the quality of life of students. The third limitation of the study was that the present study was conducted in a cross‐sectional study and among first and second year students. In order to investigate the effect of entering the university on identity threat and coping strategies, it is suggested that more studies examine the trend of entering university and engagement in the social environment of adolescents during years of university in a prospective study.

## 5 CONCLUSION

Considering the effect of mental health and that of coping strategies on the quality of life, it is recommended to evaluate the mental health of students in universities and provide training programs for identity coping in the early years of entering the university. Also, considering the effect of health literacy on the quality of life and mental health, it is suggested to screen the level of health literacy of students entering the university and provide them with the required training.

## AUTHOR CONTRIBUTIONS

SES conceived and designed the study and analyzed data. MA contributed to the study design and conducted the analysis. AJM served as the study's scientific advisor, MY collected the data, SES, MA, MY, and AJM wrote the primary draft of paper. All authors read and approved the final version of the manuscript.

## FUNDING INFORMATION

None.

## CONFLICT OF INTEREST STATEMENT

The authors declare no conflict of interest.

## ETHICAL APPROVAL

Approval of the research protocol by an Institutional Reviewer Board: The study was approved by the Ethics Committee of Alborz University of Medical Sciences with code (IR.ABZUMS.REC.1400.088).

Informed Consent: The study was conducted following the Helsinki declaration. The participants received written and oral information about the study, and written informed consent was obtained from them. They were free to decline participation or to withdraw at any time.

Registry and the Registration No. No. of the Study/Trial: N/A.

Animal Studies: Not applicable.

## Supporting information


Table S1

Table S2

## Data Availability

The data that support the findings of this study are available in supporting information.
