# Supplementary material for: Coping with identity threat and health literacy on the quality of life and mental health in students: Structural equation modeling
Source: Neuropsychopharmacol Rep. 2023 Mar 14;43(2):195–201. doi: 10.1002/npr2.12328 (PMC10275290; doi:10.1002/npr2.12328)
Supplement: Supplementary file 1 — Table S1 [file NPR2-43-195-s002.pdf]

quality of life1	quality of life2	quality of life3	quality of life4
3	3	5	4
4	5	5	5
4	5	4	4
3	2	2	5
3	3	2	2
5	4	1	3
3	4	2	2
3	4	2	2
2	3	3	4
4	4	5	5
4	5	5	5
2	3	4	5
3	4	5	5
5	5	4	5
3	4	4	5
3	4	4	5
5	5	3	3
3	5	4	5
3	4	4	5
3	3	4	5
4	3	3	4
1	2	5	2
3	3	3	3
5	5	5	5
5	5	4	5
5	4	4	5
5	3	5	5
4	5	5	4
3	4	3	3
3	4	5	5
4	4	4	4
4	5	5	5
4	4	5	5
4	3	4	4
4	5	5	5
4	4	5	4
3	3	5	3
4	3	3	4
4	5	5	5
3	3	3	5
1	2	4	4
3	3	5	5
3	4	3	5

2	2	1	3
4	4	4	2
4	5	5	5
3	5	5	5
5	3	3	3
4	5	5	5
4	3	4	4
3	4	4	4
3	3	5	5
5	5	5	5
4	4	4	4
4	5	5	5
2	4	4	1
4	3	5	5
3	4	4	4
3	4	4	5
4	5	5	4
5	4	5	5
4	4	3	4
4	4	5	5
4	5	4	5
5	4	4	3
5	5	5	5
4	5	3	3
3	3	5	5
3	4	5	5
3	3	3	4
1	2	1	4
2	3	3	5
3	4	3	2
3	4	4	4
5	5	5	5
4	4	2	1
4	5	5	5
4	5	3	5
4	5	3	5
3	4	5	5
3	3	4	4
3	3	4	3
3	3	5	5
3	4	5	5
3	4	3	4
3	5	5	5
4	4	4	5

3	4	5	4
3	4	5	5
5	4	5	4
1	3	2	3
5	5	5	5
3	4	5	5
3	4	4	5
4	5	4	5
3	4	5	5
5	5	5	5
3	4	5	5
4	4	5	5
4	4	4	5
4	5	3	5
4	5	5	5
2	5	4	5
4	4	4	5
5	5	5	5
5	5	5	5
4	5	1	2
4	5	5	5
5	4	5	5
4	5	4	1
3	3	5	5
2	3	5	5
5	5	5	5
5	5	5	5
5	5	4	5
3	3	3	3
3	3	4	5
5	4	3	3
4	4	4	5
4	3	3	5
3	3	3	3
4	4	5	4
4	4	5	4
3	3	3	2
4	5	5	5
3	4	5	5
3	4	5	5
3	3	5	2
4	5	5	4
2	3	3	4
3	4	3	4

5	5	5	5
2	2	1	5
5	5	5	5
4	4	5	5
3	3	3	4
5	5	5	5
4	4	4	5
4	5	5	5
3	3	5	5
4	5	5	5
3	3	3	4
5	5	5	5
4	5	5	5
4	4	5	4
4	4	5	5
4	5	5	5
3	4	5	5
4	3	4	4
3	4	4	4
4	3	3	1
5	4	4	5
4	3	4	5
4	5	4	5
3	3	5	4
4	5	3	5
4	5	5	4
3	2	2	5
3	3	4	2
5	4	4	3
3	4	4	2
3	4	4	2
2	3	3	4
4	4	4	5
4	5	4	5
2	3	3	5
3	4	5	5
5	5	3	5
3	4	4	5
3	4	3	5
5	5	3	3
3	5	3	5
3	4	4	5
3	3	3	5
4	3	3	4

3	3	1	3
5	5	1	5
5	5	4	5
5	4	2	5
5	3	5	5
4	5	4	4
3	4	3	3
3	4	4	5
4	4	5	4
4	5	4	5
4	4	4	5
4	3	5	4
4	5	4	5
4	4	5	4
3	3	5	3
4	3	3	4
4	5	4	5
3	3	4	5
1	2	5	4
3	3	5	5
3	4	3	5
2	2	1	3
4	4	4	2
4	5	5	5
3	5	5	5
5	3	3	3
4	5	5	5
4	3	4	4
3	4	4	4
3	3	5	5
5	5	5	5
4	4	4	4
4	5	5	5
2	4	4	1
4	3	5	5
3	4	4	4
3	4	4	5
4	5	5	4
5	4	5	5
4	4	3	4
4	4	5	5
4	5	5	5
5	4	5	3
5	5	5	5

4	5	3	3
3	3	4	5
3	4	4	5
3	3	3	4
1	2	2	4
2	3	4	5
3	4	4	2
3	4	4	4
5	5	4	5
4	4	2	1
4	5	5	5
4	5	3	5
4	5	3	5
3	4	5	5
3	3	4	4
3	3	5	3
3	3	5	5
3	4	5	5
3	4	3	4
3	5	5	5
4	4	4	5
3	4	5	4
3	4	5	5
5	4	5	4
1	3	2	3
5	5	5	5
3	4	5	5
3	4	4	5
4	5	4	5
3	4	5	5
5	5	5	5
3	4	5	5
4	4	5	5
4	4	4	5
4	5	3	5
4	5	5	5
2	5	4	5
4	4	4	5
5	5	5	5
5	5	5	5
	3		
5	4	5	5
4	5	4	1

3	3	5	5
2	3	5	5
5	5	5	5
5	5	5	5
5	5	4	5
3	3	3	3
3	3	4	5
5	4	3	3
4	4	4	5
4	3	3	5
3	3	3	3
4	4	5	4
4	4	5	4
3	3	3	2
4	5	5	5
3	4	5	5
3	4	5	5
3	3	5	2
4	5	5	4
2	3	3	4
3	4	3	4
5	5	5	5
2	2	1	5
4	5	5	4
3	4	3	3
3	3	3	4
5	5	5	5
4	5	5	5
4	4	5	4
4	4	5	5
4	5	5	5
3	4	5	5
4	3	4	4
3	4	4	4
3	4	5	5
3	4	4	5

quality of life5	quality of life6	quality of life7	quality of life8
2	4	4	3
3	3	4	4
4	4	4	4
3	2	3	2
3	2	3	2
5	5	5	5
3	3	2	3
3	3	2	3
1	1	1	2
4	4	5	4
4	4	5	4
2	3	3	2
4	4	4	4
4	5	4	5
2	1	4	3
3	3	3	2
5	5	4	4
4	4	3	3
4	5	3	3
3	3	4	4
3	4	4	5
1	5	3	1
3	3	3	3
5	5	5	5
5	5	3	4
5	5	3	4
1	1	3	3
5	5	5	5
4	4	4	3
3	3	3	4
4	4	4	4
3	3	3	3
3	3	4	5
4	3	4	4
5	5	4	5
5	5	5	5
3	4	3	2
4	4	3	4
4	4	4	4
2	2	3	2
3	4	4	3
4	3	3	4
2	3	2	2

1		1	2
4	4	4	4
2	3	3	2
2	5	3	3
3	5	3	5
5	5	4	4
4	4	4	3
3	3	1	2
3	3	4	4
5	5	3	5
4	4	4	4
3	5	5	4
4	4	4	4
4	4	5	4
3	2	3	4
3	3	3	3
4	3	4	4
4	5	4	5
5	5	5	5
4	4	3	4
4	5	4	3
5	5	4	5
5	5	5	5
4	5	4	4
2	2	2	3
3	5	5	3
3	3	2	3
1	2	1	1
3	4	3	2
4	4	3	4
3	3	4	4
5	5	5	5
3	4	4	4
5	4	4	4
3	4	4	5
3	4	4	3
3	4	4	3
2	4	4	4
3	3	5	3
3	3	3	2
2	2	3	3
4	4	2	4
3	4	2	2
4	4	4	4

2	2	2	3
3	4	2	2
4	4	4	4
1	1	3	1
5	5	4	5
4	4	4	4
4	4	4	4
4	5	2	3
4	2	4	4
4	5	5	5
4	5	4	4
4	5	4	5
4	4	3	4
4	4	3	4
4	5	5	4
1	1	2	2
4	4	4	4
5	5	4	5
5	5	4	5
3	5	5	4
5	4	4	4
4	5	4	5
4	3	3	4
1	2	2	4
2	4	3	1
5	5	5	5
5	5	4	5
5	5	5	4
3	3	3	3
3	4	4	4
4	5	4	3
4	5	5	5
3	5	4	3
3	3	3	3
4	5	4	4
4	5	4	4
3	4	3	4
5	4	3	4
3	4	4	4
3	3	4	3
1	1	3	1
4	5	4	4
3	3	3	2
3	2	4	2

4	5	4	5
1	2	3	1
5	5	5	5
3	3	5	4
2	4	3	3
5	5	5	5
4	4	5	4
5	2	2	5
4	4	3	4
5		5	5
3	3	2	4
4	5	4	5
4	4	5	4
3	5	4	4
3	5	4	5
4	5	3	5
3	4	4	5
3	3	2	3
3	4	3	3
4	4	4	4
5	5	5	5
4	5	5	5
4	4	4	3
2	4	4	3
3	3	4	4
4	4	4	4
3	2	3	2
3	2	3	2
5	5	5	5
3	3	2	3
3	3	2	3
1	1	1	2
4	4	5	4
4	4	5	4
2	3	3	2
4	4	4	4
4	5	4	5
2	1	4	3
3	3	3	2
5	5	4	4
4	4	3	3
4	5	3	3
3	3	4	4
3	4	4	5

3	3	3	3
5	5	5	5
5	5	3	4
5	5	3	4
1	1	3	3
5	5	5	5
4	4	4	3
3	3	3	4
4	4	4	4
3	3	3	3
3	3	4	5
4	3	4	4
5	5	4	5
5	5	5	5
3	4	3	2
4	4	3	4
4	4	4	4
2	2	3	2
3	4	4	3
4	3	3	4
2	3	2	2
1		1	2
4	4	4	4
2	3	3	2
2	5	3	3
3	5	3	5
5	5	4	4
4	4	4	3
3	3	1	2
3	3	4	4
5	5	3	5
4	4	4	4
3	5	5	4
4	4	4	4
4	4	5	4
3	2	3	4
3	3	3	3
4	3	4	4
4	5	4	5
5	5	5	5
4	4	3	4
4	5	4	3
5	5	4	5
5	5	5	5

4	5	4	4
2	2	2	3
3	5	5	3
3	3	2	3
1	2	1	1
3	4	3	2
4	4	3	4
3	3	4	4
5	5	5	5
3	4	4	4
5	4	4	4
3	4	4	5
3	4	4	3
3	4	4	3
2	4	4	4
3	3	5	3
3	3	3	2
2	2	3	3
4	4	2	4
3	4	2	2
4	4	4	4
2	2	2	3
3	4	2	2
4	4	4	4
1	1	3	1
5	5	4	5
4	4	4	4
4	4	4	4
4	5	2	3
4	2	4	4
4	5	5	5
4	5	4	4
4	5	4	5
4	4	3	4
4	4	3	4
4	5	5	4
1	1	2	2
4	4	4	4
5	5	4	5
5	5	4	5
4	5	4	5
4	3	3	4

1	2	2	4
2	4	3	1
5	5	5	5
5	5	4	5
5	5	5	4
3	3	3	3
3	4	4	4
4	5	4	3
4	5	5	5
3	5	4	3
3	3	3	3
4	5	4	4
4	5	4	4
3	4	3	4
5	4	3	4
3	4	4	4
3	3	4	3
1	1	3	1
4	5	4	4
3	3	3	2
3	2	4	2
4	5	4	5
1	2	3	1
5	5	5	5
4	4	4	3
3	3	2	4
4	5	4	5
4	4	5	4
3	5	4	4
3	5	4	5
4	5	3	5
3	4	4	5
3	3	2	3
3	4	3	3
4	4	4	4
4	4	4	4

quality of life9	quality of life10	quality of life11	quality of life12
3	3	5	3
5	4	4	4
4	4	4	3
4	3	1	2
4	3	3	4
4	1	4	4
4	2	3	3
4	2	3	3
4	1	2	3
4	4	4	4
3	5	4	4
2	2	4	4
3	5	5	4
5	5	4	3
5	3	2	4
4	3	4	4
4	5	4	4
3	3	3	3
5	3	4	3
4	3	4	3
5	3	3	3
4	1	4	3
3	3	3	3
5	4	4	3
5	5	4	5
5	4	5	4
3	3	3	3
5	5	5	4
5	4	4	4
4	3	3	2
4	4	4	4
3	4	5	5
5	4	4	2
4	4	5	5
4	5	5	5
5	5	5	4
2	3	1	4
4	3	4	4
4	4	4	4
3	3	4	3
4	4	4	2
4	5	4	4
5	2	2	3

3	1	1	2
4	4	4	3
5	4	1	3
5	3	5	5
5	1	5	5
4	4	5	4
4	4	5	3
5	2	3	2
4	4	4	3
5	5	5	5
4	4	4	4
5	4	3	3
5	4	4	3
5	4	4	4
4	1	3	2
4	4	4	3
3	4	4	4
5	5	1	2
5	4	5	3
4	3	3	1
3	5	5	4
4	5	4	5
5	5	5	4
4	4	5	4
4	2	4	3
4	4	4	3
4	4	5	2
1	1	3	2
2	4	4	2
3	4	4	3
4	4	3	3
5	5	5	5
4	4	3	4
4	4	3	3
5	3	5	5
5	3	4	4
5	4	4	4
4	3	4	3
4	2	4	4
4	3	4	4
4	3	4	2
5	4	3	2
4	2	4	3
5	4	4	4

5	2	3	3
3	3	4	4
5	4	4	4
3	1	2	1
4	4	5	4
4	4	5	3
5	4	4	3
4	5	4	4
4	4	3	4
5	5	5	5
4	4	4	3
5	4	4	4
5	4	4	3
5	3	3	3
5	5	5	3
5	1	3	5
5	5	5	4
5	5	5	4
4	5	5	5
5	5	3	5
4	4	3	3
4	4	5	5
5	3	2	3
5	3	4	2
4	3	4	3
5	5	5	4
5	5	5	4
4	5	5	3
3	3	3	3
4	5	5	4
4	4	3	3
5	4	5	4
4	5	4	4
3	3	3	3
5	4	4	4
5	4	4	4
4	4	2	3
5	5	4	4
4	3	4	4
5	3	4	3
4	1	3	3
4	3	4	3
4	3	3	2
5	4	4	3

5	5	4	3
1	1	1	1
5	5	5	5
4	4	5	3
3	3	4	3
5	5	5	5
3	3	3	4
4	3	2	3
4	4	4	3
5	5	5	5
3	3	4	5
4	5	5	5
3	4	4	3
3	3	4	3
4	4	5	4
5	4	5	5
4	5	3	3
3	2	3	3
4	2	4	1
3	4	4	3
4	5	4	5
4	4	3	4
4	3	4	5
3	3	5	3
5	4	4	4
4	4	4	3
4	3	1	2
4	3	3	4
4	1	4	4
4	2	3	3
4	2	3	3
4	1	2	3
4	4	4	4
3	5	4	4
2	2	4	4
3	5	5	4
5	5	4	3
5	3	2	4
4	3	4	4
4	5	4	4
3	3	3	3
5	3	4	3
4	3	4	3
5	3	3	3

3	3	3	3
5	4	4	3
5	5	4	5
5	4	5	4
3	3	3	3
5	5	5	4
5	4	4	4
4	3	3	2
4	4	4	4
3	4	5	5
5	4	4	2
4	4	5	5
4	5	5	5
5	5	5	4
2	3	1	4
4	3	4	4
4	4	4	4
3	3	4	3
4	4	4	2
4	5	4	4
5	2	2	3
3	1	1	2
4	4	4	3
5	4	1	3
5	3	5	5
5	1	5	5
4	4	5	4
4	4	5	3
5	2	3	2
4	4	4	3
5	5	5	5
4	4	4	4
5	4	3	3
5	4	4	3
5	4	4	4
4	1	3	2
4	4	4	3
3	4	4	4
5	5	1	2
5	4	5	3
4	3	3	1
3	5	5	4
4	5	4	5
5	5	5	4

4	4	5	4
4	2	4	3
4	4	4	3
4	4	5	2
1	1	3	2
2	4	4	2
3	4	4	3
4	4	3	3
5	5	5	5
4	4	3	4
4	4	3	3
5	3	5	5
5	3	4	4
5	4	4	4
4	3	4	3
4	2	4	4
4	3	4	4
4	3	4	2
5	4	3	2
4	2	4	3
5	4	4	4
5	2	3	3
3	3	4	4
5	4	4	4
3	1	2	1
4	4	5	4
4	4	5	3
5	4	4	3
4	5	4	4
4	4	3	4
5	5	5	5
4	4	4	3
5	4	4	4
5	4	4	3
5	3	3	3
5	5	5	3
5	1	3	5
5	5	5	4
5	5	5	4
4	5	5	5
4	4	5	5
5	3	2	3

5	3	4	2
4	3	4	3
5	5	5	4
5	5	5	4
4	5	5	3
3	3	3	3
4	5	5	4
4	4	3	3
5	4	5	4
4	5	4	4
3	3	3	3
5	4	4	4
5	4	4	4
4	4	2	3
5	5	4	4
4	3	4	4
5	3	4	3
4	1	3	3
4	3	4	3
4	3	3	2
5	4	4	3
5	5	4	3
1	1	1	1
5	5	5	4
5	4	4	4
3	3	4	5
4	5	5	5
3	4	4	3
3	3	4	3
4	4	5	4
5	4	5	5
4	5	3	3
3	2	3	3
4	2	4	1
4	4	5	3
5	4	4	3

quality of life13	quality of life14	quality of life15	quality of life16
3	3	4	2
4	3	3	4
4	4	3	4
5	2	3	4
4	3	3	2
4	4	4	4
5	2	2	2
5	2	2	2
4	3	2	2
4	4	4	4
5	4	3	3
4	2	1	2
5	4	4	5
5	4	5	5
5	4	1	1
3	3	2	4
3	3	3	4
3	2	3	4
5	4	3	2
3	3	2	4
4	3	3	3
4	5	2	4
3	3	3	3
4	4	4	5
5	2	4	5
3	2	2	4
5	5	3	5
5	5	4	5
4	3	2	4
4	3	3	4
4	4	4	4
4	2	2	4
3	4	3	4
5	5	5	3
4	4	4	5
5	3	5	4
3	2	1	2
4	4	2	2
4	4	4	4
4	2	1	3
4	2	2	4
5	3	3	5
4	2	2	2

3	2	1	3
4	4	4	4
5	4	2	2
5	5	1	1
3	5	5	3
4	4	3	4
5	3	3	4
5	2	2	2
4	4	3	4
5	3	2	4
4	4	4	4
4	4	2	5
4	4	3	3
4	4	4	3
4	3	3	4
3	2	2	4
3	4	5	5
3	4	4	3
5	4	4	3
4	2	2	3
4	3	2	2
5	4	4	5
5	5	5	5
5	3	3	2
3	2	3	3
4	4	2	2
4	3	2	2
4	1	1	3
4	3	2	3
3	4	2	3
3	2	3	4
5	5	5	5
4	4	4	4
4	3	4	4
3	3	2	2
4	3	2	4
5	3	3	4
4	2	3	2
2	4	4	3
4	3	3	4
3	2	2	3
4	3	1	4
5	4	2	1
4	4	4	5

4	3	3	3
3	2	2	3
4	3	4	3
5	2	1	1
2	4	4	4
5	5	4	5
4	4	4	4
5	2	3	5
4	4	4	5
5	5	4	4
3	3	3	3
5	4	3	3
3	2	4	4
3	3	3	3
5	4	5	1
3	4	1	1
5	5	5	5
5	4	5	5
4	4	4	3
5	5	4	5
4	3	4	4
5	3	4	4
4	3	3	4
4	1	2	3
4	1	1	1
4	4	3	5
4	4	4	5
5	5	5	5
3	3	3	3
5	4	3	4
3	3	4	5
5	3	3	
4	2	2	3
3	3	3	3
5	5	5	5
5	5	5	5
4	3	2	4
4	4	4	3
5	4	3	4
3	3	3	3
2	2	2	2
2	3	2	4
4	3	2	2
4	3	3	2

5	4	4	3
5	3	1	1
5	5	5	5
3	3	2	4
3	2	2	3
5	5	5	5
5	2	4	3
1	4	2	4
2	3	4	5
5	4	4	4
3	3	2	2
5	4	5	4
5	4	3	3
3	2	2	2
4	3	3	4
5	4	5	5
4	3	3	4
2	2	2	2
3	3	2	2
4	4	3	3
5	4	3	3
5	3	5	5
5	5	4	3

3	3	3	3
4	4	4	5
5	2	4	5
3	2	2	4
5	5	3	5
5	5	4	5
4	3	2	4
4	3	3	4
4	4	4	4
4	2	2	4
3	4	3	4
5	5	5	3
4	4	4	5
5	3	5	4
3	2	1	2
4	4	2	2
4	4	4	4
4	2	1	3
4	2	2	4
5	3	3	5
4	2	2	2
3	2	1	3
4	4	4	4
5	4	2	2
5	5	1	1
3	5	5	3
4	4	3	4
5	3	3	4
5	2	2	2
4	4	3	4
5	3	2	4
4	4	4	4
4	4	2	5
4	4	3	3
4	4	4	3
4	3	3	4
3	2	2	4
3	4	5	5
3	4	4	3
5	4	4	3
4	2	2	3
4	3	2	2
5	4	4	5
5	5	5	5

5	3	3	2
3	2	3	3
4	4	2	2
4	3	2	2
4	1	1	3
4	3	2	3
3	4	2	3
3	2	3	4
5	5	5	5
4	4	4	4
4	3	4	4
3	3	2	2
4	3	2	4
5	3	3	4
4	2	3	2
2	4	4	3
4	3	3	4
3	2	2	3
4	3	1	4
5	4	2	1
4	4	4	5
4	3	3	3
3	2	2	3
4	3	4	3
5	2	1	1
2	4	4	4
5	5	4	5
4	4	4	4
5	2	3	5
4	4	4	5
5	5	4	4
3	3	3	3
5	4	3	3

5	5	4	5
4	3	2	4
3	3	2	2
5	4	5	4
5	4	3	3
3	2	2	2
4	3	3	4
5	4	5	5
4	3	3	4
2	2	2	2
3	3	2	2
5	5	4	5
4	4	4	4

quality of life17	quality of life18	quality of life19	quality of life20
3	4	3	3
5	5	5	3
4	4	4	4
4	4	3	3
4	4	3	4
3	3	3	4
2	2	3	2
2	2	3	2
1	1	3	2
4	4	4	4
4	5	4	3
3	3	2	3
5	4	4	5
3	4	4	5
4	4	2	2
4	4	3	3
4	4	4	4
4	3	3	3
4	4	4	2
3	3	3	2
4	4	4	3
4	3	3	1
3	3	3	3
4	4	4	4
5	5	5	5
4	3	3	5
3	5	5	1
5	5	5	5
4	3	3	3
3	3	4	3
4	4	4	4
4	4	3	3
4	3	3	5
4	3	4	5
5	5	5	4
5	5	5	5
2	3	2	4
3	3	3	4
4	4	4	5
3	4	3	4
3	4	3	5
5	4	4	3
2	2	3	3

2	1	2	1
4	4	3	3
4	4	3	3
5	3	3	5
3	5	5	5
3	4	4	5
4	4	4	5
3	2	2	5
4	4	4	3
5	5	5	3
4	4	4	4
4	4	4	4
4	4	4	3
4	4	4	4
3	2	2	2
3	3	3	3
5	5	4	4
5	5	5	2
3	3	3	4
4	4	3	4
4	4	4	4
5	5	5	5
5	5	5	5
4	4	4	4
3	3	4	4
5	5	3	5
4	5	3	4
4	4	2	1
3	2	2	2
4	4	3	4
5	5	4	4
5	5	5	5
4	3	3	3
4	5	5	4
3	4	4	5
3	3	3	3
4	4	4	3
4	3	2	2
3	3	3	5
3	4	3	2
3	3	4	5
3	4	5	2
2	3	4	3
4	3	4	3

3	3	3	3
3	3	3	2
4	4	4	3
4	3	1	1
4	4	5	4
4	4	3	4
4	4	4	4
4	5	4	3
4	4	4	2
	5	5	5
4	4	3	4
5	5	4	4
4	4	4	4
2	3	3	3
4	5	3	5
1	1	3	3
5	5	5	5
5	5	5	4
5	5	5	5
5	3	5	5
4	5	5	4
5	5	5	5
3	3	3	3
4	4	3	4
3	3	3	3
5	5	5	5
5	5	5	5
5	5	5	4
3	3	3	3
4	4	4	5
4	4	4	5
5	5	5	5
3	4	3	4
3	3	3	3
4	5	5	3
4	5	5	3
4	4	4	3
5	5	5	4
4	4	4	3
3	3	4	4
2	2	2	1
4	4	5	4
3	3	3	2
3	3	4	3

3	4	5	5
2	4	1	1
5	5	5	5
4	5	4	4
3	3	3	2
5	5	5	5
4	4	4	3
3	3	4	5
3	3	4	2
4	5	5	4
2	3	4	4
5	5	5	4
4	5	3	5
3	4	3	4
5	4	5	4
5	5	5	4
4	5	4	4
3	3	3	2
3	3	3	2
4	2	3	4
4	4	4	3
5	5	5	3
2	4	4	4

3	3	3	3
4	4	4	4
5	5	5	5
4	3	3	5
3	5	5	1
5	5	5	5
4	3	3	3
3	3	4	3
4	4	4	4
4	4	3	3
4	3	3	5
4	3	4	5
5	5	5	4
5	5	5	5
2	3	2	4
3	3	3	4
4	4	4	5
3	4	3	4
3	4	3	5
5	4	4	3
2	2	3	3
2	1	2	1
4	4	3	3
4	4	3	3
5	3	3	5
3	5	5	5
3	4	4	5
4	4	4	5
3	2	2	5
4	4	4	3
5	5	5	3
4	4	4	4
4	4	4	4
4	4	4	3
4	4	4	4
3	2	2	2
3	3	3	3
5	5	4	4
5	5	5	2
3	3	3	4
4	4	3	4
4	4	4	4
5	5	5	5
5	5	5	5

4	4	4	4
3	3	4	4
5	5	3	5
4	5	3	4
4	4	2	1
3	2	2	2
4	4	3	4
5	5	4	4
5	5	5	5
4	3	3	3
4	5	5	4
3	4	4	5
3	3	3	3
4	4	4	3
4	3	2	2
3	3	3	5
3	4	3	2
3	3	4	5
3	4	5	2
2	3	4	3
4	3	4	3
3	3	3	3
3	3	3	2
4	4	4	3
4	3	1	1
4	4	5	4
4	4	3	4
4	4	4	4
4	5	4	3
4	4	4	2
	5	5	5
4	4	3	4
5	5	4	4

5	5	5	5
4	3	3	3
2	3	4	4
5	5	5	4
4	5	3	5
3	4	3	4
5	4	5	4
5	5	5	4
4	5	4	4
3	3	3	2
3	3	3	2
4	4	3	4
4	4	4	4

quality of life21	quality of life22	quality of life23	quality of life24
2	2	2	4
	4	4	5
3	4	4	4
4	2	3	4
2	5	4	4
5	5	4	5
4	2	3	3
4	2	3	3
3	4	4	4
4	4	4	4
2	4	4	5
4	4	2	3
	3	4	4
3	4	5	5
3	3	5	5
3	2	4	4
	3	5	5
	4	4	3
	3	4	4
3	3		3
2	2	3	4
2	3	3	3
3	3	3	3
3	4	4	5
	5	5	5
4	5	5	4
	4	5	5
	5	4	5
3	3	4	4
5	4	3	3
4	4	4	4
	4	3	3
	2	3	3
2	4	4	4
	4	5	4
4	4	5	5
1	3	4	5
4	4	4	4
5	3	3	4
3	4	3	3
4	3	3	3
4	3	3	3
3	2	5	5

1	4	2	3
3	3	4	4
1	2	1	3
1	3	3	5
5	5	5	5
	5	4	3
3	3	4	5
	5	3	5
3	3	4	3
3	2	5	5
3	4	4	4
4	3	5	5
4	4	4	4
	4	4	5
	4	3	4
1	2	4	4
3	5	4	4
1	2	2	5
5	4	5	4
1	2	1	2
3	4	3	3
	5	5	5
3	4	4	5
	3	3	5
3	1	4	4
4	4	3	4
4	4	4	3
1	1	1	2
3	3	3	2
2	4	3	3
5	2	4	5
3	5	5	5
3	4	4	4
1	4	4	4
2	5	4	4
3	3	4	4
	3	3	5
	4	4	3
4	4	2	4
3	3	4	3
4	2	4	5
	3	2	3
	4	2	4
4	4	4	4

1	3	3	3
1	2	1	3
	5	5	5
1	1	1	1
	4	4	4
1	4	4	5
3	3	3	4
1	4	5	5
3	4	3	2
	5	5	5
4	4	3	3
4	5	4	4
2	3	3	5
3	2	4	4
1	4	1	3
3	2	5	5
4	5	5	5
	4	4	5
	5	5	5
3	4	4	4
1	4	4	4
1	4	4	5
2	4	4	5
	4	4	4
5	4	1	1
5	1	5	5
5	5	4	4
1	4	4	5
3	3	3	3
4	4	5	4
5	4	3	4
5	4	4	5
4	4	4	4
3	3	3	3
3	5	4	5
3	5	4	5
1	4	3	4
2	5	5	5
	4	5	5
3	4	4	3
1	1	3	3
1	4	5	5
3	3	2	4
3	2	5	4

1	3	4	5
1	3	1	2
5	5	5	4
4	4	3	4
1	3	2	2
5	5	5	5
5	4	4	5
3	4	3	4
2	4	1	4
4	4	5	5
	3	2	2
	5	3	5
3	2	3	3
5	3	3	3
4	5	4	5
5	4	4	5
	3	3	4
2	2	3	3
	2	1	2
4	3	3	2
1	3	4	4
3	3	5	5
4	3	3	3

3	3	3	3
3	4	4	5
	5	5	5
4	5	5	4
	4	5	5
	5	4	5
3	3	4	4
5	4	3	3
4	4	4	4
	4	3	3
	2	3	3
2	4	4	4
	4	5	4
4	4	5	5
1	3	4	5
4	4	4	4
5	3	3	4
3	4	3	3
4	3	3	3
4	3	3	3
3	2	5	5
1	4	2	3
3	3	4	4
1	2	1	3
1	3	3	5
5	5	5	5
	5	4	3
3	3	4	5
	5	3	5
3	3	4	3
3	2	5	5
3	4	4	4
4	3	5	5
4	4	4	4
	4	4	5
	4	3	4
1	2	4	4
3	5	4	4
1	2	2	5
5	4	5	4
1	2	1	2
3	4	3	3
	5	5	5
3	4	4	5

	3	3	5
3	1	4	4
4	4	3	4
4	4	4	3
1	1	1	2
3	3	3	2
2	4	3	3
5	2	4	5
3	5	5	5
3	4	4	4
1	4	4	4
2	5	4	4
3	3	4	4
	3	3	5
	4	4	3
4	4	2	4
3	3	4	3
4	2	4	5
	3	2	3
	4	2	4
4	4	4	4
1	3	3	3
1	2	1	3
	5	5	5
1	1	1	1
	4	4	4
1	4	4	5
3	3	3	4
1	4	5	5
3	4	3	2
	5	5	5
4	4	3	3
4	5	4	4

	5	4	5
3	3	4	4
	3	2	2
	5	3	5
3	2	3	3
5	3	3	3
4	5	4	5
5	4	4	5
	3	3	4
2	2	3	3
	2	1	2
1	4	4	5
3	3	3	4

quality of life25	quality of life26	healthlitracy1	healthlitracy2
3	4	4	4
5	3	5	5
4	4	3	4
4	3	5	5
3	2	3	5
5	5	5	5
3	2	5	4
3	2	5	4
4	1	3	2
4	2	5	5
5	3	4	3
4	2	4	4
3	4		
5	5	5	5
5	1	5	5
4	3	3	3
4	4	3	3
3	4	4	3
4	1	2	2
3	3	3	3
3	2	5	5
2	1		
3	3	3	3
4	3	4	4
5	5	1	3
3	2	3	4
3	1	5	5
5	5	5	5
4	3	5	4
3	3	5	5
4	4	4	3
2	2	4	4
1	3	5	3
2	3	5	5
4	4	3	5
3	4	5	5
5	4	5	5
5	4	5	5
3	3	5	5
4	3	1	2
2	3	4	4
5	4	3	4
5	4	4	4

3	2	3	3
4	3	4	4
1	1	5	5
5	2	4	5
5	3	5	5
4	2	5	5
5	4	3	4
5	1	3	5
3	4	4	4
5	3	5	3
4	4	5	4
5	3	5	5
3	3	4	4
4	4	4	4
3	1	4	4
4	3	4	4
2	4	5	5
2	4	1	1
4	4	5	5
1	3	3	3
4	2	5	3
4	5	5	5
4	3	4	4
4	3	5	5
4	1	3	3
3	2	5	4
3	3	4	4
1	1	4	4
2	3	2	3
1	3	3	4
5	2		
5	5	3	5
3	2	4	4
4	4	3	3
5	2	4	3
3	3	5	4
5	4	5	5
4	1	5	5
4	4	4	4
3	2	4	5
5	2	3	4
2	3	2	4
3	4	3	5
4	3	4	4

3	1	4	5
2	2	5	5
5	3	5	3
1	1	5	5
4	4	4	
3	3	5	5
4	3	4	4
4	3	4	4
1	4	3	5
5	4	5	5
3	4	4	4
5	3	4	4
4	4	5	5
4	3	4	4
1	4	5	5
5	1	5	5
5	3	5	5
4	4	4	5
5	4	5	5
3	2	4	4
4	4	3	3
5	4	5	5
4	3	5	5
3	1	2	4
3	2	4	4
5	5	5	5
3	4	4	4
3	4	5	5
3	3	3	3
5	4	5	5
4	2	5	5
4	4	5	5
4	3	5	4
3	3	3	3
4	3	5	5
4	3	5	5
4	4	4	4
5	4	3	4
5	4	4	4
3	3	3	4
3	1	5	5
5	3	4	3
2	2	4	4
4	3	4	5

4	3	5	5
1	1	5	5
5	5	5	5
4	2	5	5
3	3	3	3
5	5		
4	3	4	5
2	1	2	5
5	3	5	4
5	3		
2	3	2	4
4	5	5	5
4	4	5	5
3	4	4	4
5	2	5	5
5	4	5	5
3	3	4	4
4	3	4	5
1	2	5	4
3	3	5	5
4	4	4	5
3	5	5	5
4	2	4	3

3	3	3	3
4	3	4	4
5	5	1	3
3	2	3	4
3	1	5	5
5	5	5	5
4	3	5	4
3	3	5	5
4	4	4	3
2	2	4	4
1	3	5	3
2	3	5	5
4	4	3	5
3	4	5	5
5	4	5	5
5	4	5	5
3	3	5	5
4	3	1	2
2	3	4	4
5	4	3	4
5	4	4	4
3	2	3	3
4	3	4	4
1	1	5	5
5	2	4	5
5	3	5	5
4	2	5	5
5	4	3	4
5	1	3	5
3	4	4	4
5	3	5	3
4	4	5	4
5	3	5	5
3	3	4	4
4	4	4	4
3	1	4	4
4	3	4	4
2	4	5	5
2	4	1	1
4	4	5	5
1	3	3	3
4	2	5	3
4	5	5	5
4	3	4	4

4	3	5	5
4	1	3	3
3	2	5	4
3	3	4	4
1	1	4	4
2	3	2	3
1	3	3	4
5	2		
5	5	3	5
3	2	4	4
4	4	3	3
5	2	4	3
3	3	5	4
5	4	5	5
4	1	5	5
4	4	4	4
3	2	4	5
5	2	3	4
2	3	2	4
3	4	3	5
4	3	4	4
3	1	4	5
2	2	5	5
5	3	5	3
1	1	5	5
4	4	4	
3	3	5	5
4	3	4	4
4	3	4	4
1	4	3	5
5	4	5	5
3	4	4	4
5	3	4	4

5	5	5	5
4	3	5	4
2	3	2	4
4	5	5	5
4	4	5	5
3	4	4	4
5	2	5	5
5	4	5	5
3	3	4	4
4	3	4	5
1	2	5	4
3	3	5	5
4	3	4	4

healthlitracy3	healthlitracy4	healthlitracy5	healthlitracy6
4	4	5	5
4	5	5	5
4	5	4	4
3	5	5	5
5	5	5	5
5	5	5	5
4	5	5	5
4	5	5	5
2	3	2	4
5	5	5	5
4	4	4	5
5	5	4	4
5	5	5	5
4	4	4	3
2	3	4	4
5	5	5	5
3	3	3	3
2	3	5	4
3	3	4	4
5	5	5	5
3	3	3	3
3	4	4	4
4	4	4	4
4	4	5	5
5	5	5	5
5	5	5	5
4	5	5	5
5	5	5	5
4	3	4	4
4	4	4	4
3	3	4	4
5	5	5	5
5	5	5	5
5	5	5	5
5	5	5	5
5	5	4	3
5	5	5	5
1	2	2	3
4	4	4	3
4	4	4	5
4	3	4	5

2	4	4	4
4	4	4	4
5	3	5	5
5	5	5	5
5	5	5	5
5	5	5	5
4	3	5	5
5	5	5	5
4	4	4	5
5	5	5	5
5	4	4	4
5	5	5	5
4	5	5	4
4	5	4	5
5	5	4	4
4	4	4	4
5	5	5	5
1	1	3	5
5	5	5	5
3	3	4	4
5	5	5	5
5	5	5	5
4	5	5	5
3	4	5	5
3	3	4	4
4	4	5	4
4	4	4	4
4	4	5	5
3	3	3	2
3	3	4	4
3	3	4	4
5	4	5	5
5	5	4	4
4	4	4	4
4	5	5	5
5	5	5	5
5	5	5	5
4	3	3	4
5	5	3	3
4	3	4	4
5	5	4	5
5	5	5	5
4	4	4	4

4	3	5	5
5	5	5	5
4	4	4	4
5	5	5	3
5	5	5	5
5	4	4	4
3	3	4	5
5	5	5	5
5	5	5	5
4	4	5	5
4	4	5	4
5	5	5	5
3	3	3	4
5	5	5	5
5	5	5	5
5	5	5	5
5	5	5	5
5	5	5	5
5	4	5	5
5	5	4	4
5	5	5	5
5	5	5	5
4	4	5	5
4	4	5	5
5	5	5	5
5	5	5	5
5	5	5	5
3	3	3	5
5	5	5	5
4	5	4	5
5	5	5	5
4	4	5	5
3	3	3	3
5	5	5	5
5	5	5	5
4	4	4	4
3	4	4	4
4	4	4	4
3	3	3	3
5	5	5	5
4	5	4	4
4	4	3	4
5	5	4	5

5	5	5	5
4	4	5	5
5	5	5	5
5	5	5	5
3	3	3	3
5	5	5	5
5	5	5	5
3	3	3	4
5	5	5	5
5	5	5	5
5	5	5	5
4	4	5	4
5	5	5	5
5	4	5	5
3	4	4	4
5	5	5	5
5	5	4	3
5	5	5	5
5	5	5	5
5	5	5	5
3	5	3	4

3	3	3	3
3	4	4	4
4	4	4	4
4	4	5	5
5	5	5	5
5	5	5	5
4	5	5	5
5	5	5	5
4	3	4	4
4	4	4	4
3	3	4	4
5	5	5	5
5	5	5	5
5	5	5	5
5	5	5	5
5	5	4	3
5	5	5	5
1	2	2	3
4	4	4	3
4	4	4	5
4	3	4	5
2	4	4	4
4	4	4	4
5	3	5	5
5	5	5	5
5	5	5	5
5	5	5	5
4	3	5	5
5	5	5	5
4	4	4	5
5	5	5	5
5	4	4	4
5	5	5	5
4	5	5	4
4	5	4	5
5	5	4	4
4	4	4	4
5	5	5	5
1	1	3	5
5	5	5	5
3	3	4	4
5	5	5	5
5	5	5	5
4	5	5	5

3	4	5	5
3	3	4	4
4	4	5	4
4	4	4	4
4	4	5	5
3	3	3	2
3	3	4	4
3	3	4	4
5	4	5	5
5	5	4	4
4	4	4	4
4	5	5	5
5	5	5	5
5	5	5	5
4	3	3	4
5	5	3	3
4	3	4	4
5	5	4	5
5	5	5	5
4	4	4	4
4	3	5	5
5	5	5	5
4	4	4	4
5	5	5	3
5	5	5	5
5	4	4	4
3	3	4	5
5	5	5	5
5	5	5	5
4	4	5	5
4	4	5	4

5	5	5	5
4	5	5	5
5	5	5	5
5	5	5	5
5	5	5	5
4	4	5	4
5	5	5	5
5	4	5	5
3	4	4	4
5	5	5	5
5	5	4	3
5	5	5	5
5	4	4	4

healthlitracy7	healthlitracy8.	healthlitracy9	healthlitracy10
3	3	4	5
5	5	5	5
4	4	4	4
4	5	5	5
2	5	5	5
5	5	5	5
5	5	5	5
5	5	5	5
3	3	3	3
4	4	5	5
3	4	5	5
4	4	4	5
5	5	5	5
4	4	4	4
4	3	4	4
5	5	5	5
4	3	4	5
3	5	5	4
4	4	4	4
5	4	5	5
3	3	3	3
4	4	4	4
5	5	5	5
4	4	4	5
5	5	5	5
5	5	5	5
5	5	5	5
4	5	5	5
4	4	4	4
4	4	4	5
4	3	3	5
5	5	5	5
5	5	5	5
5	5	5	5
4	5	5	5
4	4	5	5
5	5	5	5
3	3	3	
4	3	3	5
5	5	5	5
3	3	4	4

4	4	3	4
3	4	4	4
5	5	5	5
5	5	5	5
5	5	5	5
5	5	5	5
4	5	4	4
5	5	5	5
5	5	4	4
5	5	5	5
4	4	4	4
5	5	5	5
4	4	4	4
4	4	4	4
3	5	5	5
4	4	4	4
5	5	5	5
3	3	4	5
5	5	5	5
4	4	4	4
4	4	5	5
4	5	5	5
5	5	5	5
5	5	5	5
2	4	4	4
4	4	4	5
4	4	4	5
5	5	5	5
2	2	2	2
4	3	3	5
4	5	5	4
5	4	4	4
3	3	4	4
4	5	4	5
5	4	5	5
5	5	5	5
5	5	5	5
3	4	3	4
2	3	5	5
4	4	4	4
4	5	5	5
5	5	5	5
4	4	4	4

5	5	5	5
5	5	5	5
4	4	4	4
1	3	3	3
5	5	5	5
4	2	4	4
4	5	5	5
4	4	5	5
5	5	5	5
5	4	5	5
4	4	5	5
5	5	5	5
4	3	3	4
5	5	4	5
5	5	5	5
5	5	5	5
5	5	5	5
5	5	5	5
5	4	4	4
3	3	4	4
5	5	5	5
5	5	5	5
4	4	4	5
5	5	5	5
5	5	5	5
5	5	5	5
5	5	5	5
3	4	3	3
5	5	5	5
4	5	5	5
5	5	5	5
5	5	5	5
3	3	3	3
4	4	4	5
4	4	4	5
4	4	4	4
4	4	4	5
4	4	4	4
4	3	4	3
5	5	5	5
5	4	4	5
4	3	3	4
2	4	4	4

5	5	5	5
5	5	5	5
5	5	5	5
5	5	5	5
2	3	3	3
4	4	5	5
5	5	5	5
3		3	4
3		5	5
5	5	5	5
4	4	5	5
4	4	4	4
5	5	5	5
4	5	5	5
4	4	5	5
2	5	5	5
4	4	5	5
4	5	5	5
5	5	5	5
5	5	5	5
4	3	3	5

3	3	3	3
4	4	4	4
5	5	5	5
4	4	4	5
5	5	5	5
5	5	5	5
5	5	5	5
4	5	5	5
4	4	4	4
4	4	4	5
4	3	3	5
5	5	5	5
5	5	5	5
5	5	5	5
4	5	5	5
4	4	5	5
5	5	5	5
3	3	3	
4	3	3	5
5	5	5	5
3	3	4	4
4	4	3	4
3	4	4	4
5	5	5	5
5	5	5	5
5	5	5	5
5	5	5	5
4	5	4	4
5	5	5	5
5	5	4	4
5	5	5	5
4	4	4	4
5	5	5	5
4	4	4	4
4	4	4	4
3	5	5	5
4	4	4	4
5	5	5	5
3	3	4	5
5	5	5	5
4	4	4	4
4	4	5	5
4	5	5	5
5	5	5	5

5	5	5	5
2	4	4	4
4	4	4	5
4	4	4	5
5	5	5	5
2	2	2	2
4	3	3	5
4	5	5	4
5	4	4	4
3	3	4	4
4	5	4	5
5	4	5	5
5	5	5	5
5	5	5	5
3	4	3	4
2	3	5	5
4	4	4	4
4	5	5	5
5	5	5	5
4	4	4	4
5	5	5	5
5	5	5	5
4	4	4	4
1	3	3	3
5	5	5	5
4	2	4	4
4	5	5	5
4	4	5	5
5	5	5	5
5	4	5	5
4	4	5	5

5	5	5	5
5	5	5	5
3		5	5
5	5	5	5
4	4	5	5
4	4	4	4
5	5	5	5
4	5	5	5
4	4	5	5
2	5	5	5
4	4	5	5
5	5	5	5
4	2	4	4

healthlitracy11	healthlitracy12	healthlitracy13	healthlitracy14
5	5	5	5
5	5	5	5
4	4	4	4
5	5	5	5
3	5	5	5
5	5	5	5
5	5	5	5
5	5	5	5
4	5	5	5
5	5	5	5
5	5	5	5
5	5	4	4
5	5	5	5
4	4	4	4
4	4	3	3
5	5	5	5
5	5	5	5
4	3	2	2
4	4	4	5
5	5	5	5
4	4	4	5
4	4	3	4
5	5	5	5
4	5	5	5
5	5	5	5
5	5	5	3
5	5	5	4
5	5	5	5
4	4	4	4
5	5	4	4
4	4	3	4
5	5	5	4
5	5	5	5
5	5	5	5
5	5	5	5
5	5	5	5
5	5	5	5
4	4	5	5
4	4	5	5
5	5	5	5
4	4	4	5

3	3	5	4
4	4	4	4
5	5	5	5
5	5	5	5
5	5	5	5
5	5	5	5
4	5	4	5
5	5	5	5
5	4	4	4
5	5	5	5
4	4	4	4
5	5	5	5
4	3	4	3
5	5	5	5
5	5	5	4
4	4	4	4
5	5	5	5
5	4	2	5
5	5	5	5
4	4	4	4
5	5	4	5
5	5	5	5
5	5	5	5
5	5	5	5
4	4	4	4
5	5	5	5
5	5	4	5
5	5	4	4
3	3	3	4
4	3	3	
5	5	3	3
4	4	4	5
4	4	4	4
5	5	4	5
5	5	5	4
5	5	5	5
5	5	4	5
4	3	5	4
4	5	5	4
4			5
5	5	5	5
5	5	5	5
3	4	5	5

5	5	5	5
5	5	5	5
4	4	4	4
3	4	5	5
5	5	5	5
4	4	4	5
5	5	5	5
5	5	5	5
5	5	5	4
4	5	4	4
5	5	4	5
5	5	5	5
4	4	3	4
5	5	5	3
4	5	5	5
5	5	5	5
5	5	5	5
5	5	5	5
4	4	4	5
4	4	4	4
5	5	5	5
5	5	5	5
5	5	5	5
5	5	5	5
5	5	5	5
5	5	5	5
5	5	5	5
4	3	3	3
5	5	5	5
5	5	5	4
5	5	5	5
5	5	5	5
3	3	3	3
5	5	5	5
5	5	5	5
3	4	3	3
4	4	3	4
4	3	4	4
4	3	4	4
5	5	5	5
5	5	5	4
4	4	4	4
5	5	5	5

5	5	5	5
5	5	4	5
5	5	5	5
5	5	5	5
3	3	3	3
5	5	5	5
5	5	5	5
4	4	4	5
5	5	5	5
5	5	5	5
5	5	5	5
4	4	4	4
5	5	5	5
5	5	5	5
5	5	4	5
5	5	5	5
5	5	5	4
5	5	5	5
5	5	5	5
5	5	5	5
3	4	3	4

4	4	4	5
4	4	3	4
5	5	5	5
4	5	5	5
5	5	5	5
5	5	5	3
5	5	5	4
5	5	5	5
4	4	4	4
5	5	4	4
4	4	3	4
5	5	5	4
5	5	5	5
5	5	5	5
5	5	5	5
5	5	5	5
5	5	5	5
4	4	5	5
4	4	5	5
5	5	5	5
4	4	4	5
3	3	5	4
4	4	4	4
5	5	5	5
5	5	5	5
5	5	5	5
5	5	5	5
4	5	4	5
5	5	5	5
5	4	4	4
5	5	5	5
4	4	4	4
5	5	5	5
4	3	4	3
5	5	5	5
5	5	5	4
4	4	4	4
5	5	5	5
5	4	2	5
5	5	5	5
4	4	4	4
5	5	4	5
5	5	5	5
5	5	5	5

5	5	5	5
4	4	4	4
5	5	5	5
5	5	4	5
5	5	4	4
3	3	3	4
4	3	3	

5	5	3	3
4	4	4	5
4	4	4	4
5	5	4	5
5	5	5	4
5	5	5	5
5	5	4	5
4	3	5	4
4	5	5	4
4			5
5	5	5	5
5	5	5	5
3	4	5	5
5	5	5	5
5	5	5	5
4	4	4	4
3	4	5	5

5	5	5	5
4	4	4	5
5	5	5	5
5	5	5	5
5	5	5	4
4	5	4	4
5	5	4	5

5	5	5	3
5	5	5	4
5	5	5	5
5	5	5	5
5	5	5	5
4	4	4	4
5	5	5	5
5	5	5	5
5	5	4	5
5	5	5	5
5	5	5	4
5	5	5	5
4	4	4	5

healthlitracy15	healthlitracy16	healthlitracy17	healthlitracy18
5	5	5	5
5	5	5	5
5	4	5	4
5	4	5	5
5	4	5	3
5	5	5	5
5	5	1	5
5	5	1	5
5	4	5	4
5	5	1	5
5	5	5	4
5	3	5	5
5	5	5	5
4	4	4	4
4	4		4
5	3	1	5
4	3	3	3
4	1	2	4
5	4	4	4
5	5	5	5
5	4	5	4
4	4	4	4
5	5	5	5
5	4	4	5
5	5	5	5
5	5	5	5
5	5	1	5
5	5	5	5
4	4	4	4
4	4	2	4
5	4		2
5	4	1	4
5	5	5	5
5	5	5	5
5	5	5	3
5	5	4	4
5	5	5	5
5	5	5	5
5	5	5	5
5	4	4	4
5	4	3	4

5	4	4	4
4	4	4	5
5	3	4	5
5	5	5	3
5	5	5	5
5	5	5	5
5	4	4	5
5	5	5	5
4	3	2	5
5	5	5	5
4	4	4	4
5	5	1	5
4	4	4	3
4	4	4	4
5	4	5	4
4	4	4	4
5	5	5	5
5	2	1	3
5	5	5	5
4	3	4	4
5	4	2	3
5	5	5	5
5	5	5	5
5	5	3	4
4			
4	4	4	4
4	5	4	3
5	4	4	3
4	4	5	2
4	3	4	3
5	2	3	4
4	4	4	4
4	5	2	4
5	5	5	4
5	4	5	5
5	5	1	5
5	4	4	5
3	3	4	3
5	4	4	3
5	4	4	4
5	5	5	5
5	5	5	5
4	4	4	3

5	5	5	5
5	5	5	5
4	3	4	4
5	5	5	4
5	5	5	5
5	5	4	3
5	5	5	5
5	4	4	5
5	5	5	5
5	5	5	4
5	4	4	3
5	5	1	4
3	3	3	3
5	5	5	5
5	5	5	5
5	5	5	5
5	5	5	5
5	4	1	4
4	4	4	4
4	5	2	4
5	5	5	5
5	5	1	5
5	5	5	5
5	5	5	5
5	5	5	5
5	5	5	4
5	5	5	5
3	3	3	3
5	5	5	5
5	4	5	4
5	4	5	5
5	5	5	5
3	3	3	3
5	5	3	4
5	5	3	4
5	4	3	4
5	4	4	5
3	4	3	3
4	4	4	4
5	5	1	5
5	4	4	4
4	3	4	4
5	5	5	5

5	5	1	5
5	4	1	3
5	5	5	5
5	5	5	5
3	3	3	4
5	5	5	4
5	5	5	4
5	4	1	4
5	5	5	4
5	5	5	5
5	5	5	5
5	4	4	4
5	5	5	4
5	5	5	5
5	4	4	5
5	5	5	5
5	4	5	3
5	5	5	4
5	5	5	5
5	5	5	5
4	4	4	4

5	4	5	4
4	4	4	4
5	5	5	5
5	4	4	5
5	5	5	5
5	5	5	5
5	5	1	5
5	5	5	5
4	4	4	4
4	4	2	4
5	4		2
5	4	1	4
5	5	5	5
5	5	5	5
5	5	5	3
5	5	4	4
5	5	5	5
5	5	5	5
5	5	5	5
5	4	4	4
5	4	3	4
5	4	4	4
4	4	4	5
5	3	4	5
5	5	5	3
5	5	5	5
5	5	5	5
5	4	4	5
5	5	5	5
4	3	2	5
5	5	5	5
4	4	4	4
5	5	1	5
4	4	4	3
4	4	4	4
5	4	5	4
4	4	4	4
5	5	5	5
5	2	1	3
5	5	5	5
4	3	4	4
5	4	2	3
5	5	5	5
5	5	5	5

5	5	3	4
4			
4	4	4	4
4	5	4	3
5	4	4	3
4	4	5	2
4	3	4	3
5	2	3	4
4	4	4	4
4	5	2	4
5	5	5	4
5	4	5	5
5	5	1	5
5	4	4	5
3	3	4	3
5	4	4	3
5	4	4	4
5	5	5	5
5	5	5	5
4	4	4	3
5	5	5	5
5	5	5	5
4	3	4	4
5	5	5	4
5	5	5	5
5	5	4	3
5	5	5	5
5	4	4	5
5	5	5	5
5	5	5	4
5	4	4	3

5	5	5	5
5	5	1	5
5	5	5	4
5	5	5	5
5	5	5	5
5	4	4	4
5	5	5	4
5	5	5	5
5	4	4	5
5	5	5	5
5	4	5	3
5	5	5	5
5	5	4	3

healthlitracy19	healthlitracy20	healthlitracy21	healthlitracy22
5	5	5	5
5	5	5	5
3	4	4	4
4	5	5	5
3	3	5	5
5	5	5	5
5	5	5	4
5	5	5	4
4	3	3	3
5	5	5	5
4	4	4	5
4	3	5	4
5	5	5	5
4	4	2	3
4	4	4	4
4	4	3	4
5	4	3	5
4	4		3
4	4	4	4
5	5	5	5
4	4	4	4
4	4	4	4
5	5	5	5
5	5	5	5
5	5	5	5
5	5	5	5
4	5	5	5
5	5	5	5
4	4	4	4
4	4	4	4
3	3	5	3
5	4	4	4
5	5	5	5
5	5	5	5
4	5	5	5
5	5	5	5
5	4	4	5
5	5	4	5
4	5	5	5
3	3	4	3
4	4	4	4

4	4	4	4
5	4	4	4
5	5	5	4
3	3	5	3
5	5	5	5
5	5	5	5
4	4	5	3
5	5	5	5
4	4	4	4
5	5	5	5
4	4	4	4
5	5	5	5
4	3	4	4
4	4	4	4
4	4	3	4
4	4	4	4
5	5	5	5
4	5	5	4
5	5	5	5
3	4	3	4
3	4	5	4
5	5	5	5
5	5	5	5
4	5	5	5

4	4	4	4
4	4	5	4
3	3	5	4
3	3	3	2
4	5	5	3

4	5	4	5
3	4	5	4
4	4	4	3
4	4	5	4
4	5	4	4
5	5	5	5
4	4	5	5
5	4	3	2
3	4	4	2
3	4	4	4
4	3	5	5
4	4	5	3
4	3	4	3

5	5	3	4
5	5	5	5
4	4	4	4
1	3	4	4
5	4	5	5
4	4	4	3
5	5	5	5
5	5	5	2
4	5	5	5
5	5	5	5
5	4	5	4
4	4	4	5
3	3	3	4
5	5	5	5
5	5	3	4
5	5	5	5
5	5	5	4
4	4	4	5
3	4	5	4
4	4	4	3
5	5	5	5
5	5	4	4
5	5	5	5
5	5	4	5
5	5	5	5
5	5	5	5
5	5	5	5
3	3	4	3
5	5	5	5
5	5	5	5
5	5	5	5
5	5	5	5
3	3	3	3
5	5	5	4
5	5	5	4
4	4	4	4
4	4	4	4
3	3	4	4
3	4	4	4
5	5	5	5
4	4	4	4
3	4	3	3
5	5	2	4

5	5	5	5
4	5	5	5
5	5	5	5
5	5	5	4
3	3	3	3
4	5	5	4
4	5	5	2
4	4	4	4
3	4	5	4
5	5	5	5
5	5	5	5
4	4	4	4
3	4	5	5
5	5	5	4
5	5	5	4
3	4	4	4
4	4	4	4
5	5	5	4
5	5	5	5
5	5	5	5
3	5	3	4

4	4	4	4
4	4	4	4
5	5	5	5
5	5	5	5
5	5	5	5
5	5	5	5
4	5	5	5
5	5	5	5
4	4	4	4
4	4	4	4
3	3	5	3
5	4	4	4
5	5	5	5
5	5	5	5
4	5	5	5
5	5	5	5
5	4	4	5
5	5	4	5
4	5	5	5
3	3	4	3
4	4	4	4
4	4	4	4
5	4	4	4
5	5	5	4
3	3	5	3
5	5	5	5
5	5	5	5
4	4	5	3
5	5	5	5
4	4	4	4
5	5	5	5
4	4	4	4
5	5	5	5
4	3	4	4
4	4	4	4
4	4	3	4
4	4	4	4
5	5	5	5
4	5	5	4
5	5	5	5
3	4	3	4
3	4	5	4
5	5	5	5
5	5	5	5

4	5	5	5
4	4	4	4
4	4	5	4
3	3	5	4
3	3	3	2
4	5	5	3
4	5	4	5
3	4	5	4
4	4	4	3
4	4	5	4
4	5	4	4
5	5	5	5
4	4	5	5
5	4	3	2
3	4	4	2
3	4	4	4
4	3	5	5
4	4	5	3
4	3	4	3
5	5	3	4
5	5	5	5
4	4	4	4
1	3	4	4
5	4	5	5
4	4	4	3
5	5	5	5
5	5	5	2
4	5	5	5
5	5	5	5
5	4	5	4

5	5	5	5
4	5	5	5
3	4	5	4
5	5	5	5
5	5	5	5
4	4	4	4
3	4	5	5
5	5	5	4
5	5	5	4
3	4	4	4
4	4	4	4
5	4	5	5
4	4	4	3

healthlitracy23	healthlitracy24	healthlitracy25	healthlitracy26
4	4	3	4
5	5	5	5
4	3	3	3
5	2	2	3
5	5	5	5
5	4	5	4
3	2	1	5
3	2	1	5
2	1	1	2
5	5	5	5
5	3	3	5
4	5	4	4
5	5	3	3
5	5	2	3
3	2	4	3
4	5	4	4
4	3	4	4
3	2	4	4
5	5	5	5
4	3	3	5
4	4	5	5
4	5	4	4
5	5	5	5
4	2	2	3
5	2	5	5
3	4	5	3
5	5	5	5
5	5	5	5
4	4	4	4
4	4	5	3
4	3	4	4
2	3	3	4
5	5	5	5
5	5	5	5
1	1	1	3
4	3	5	5
5	4	5	4
5	5	5	5
5	5	4	4
5	3	4	1
3	3	4	3

4	4	2	3
4	4	4	4
3	3	3	3
5	3	3	4
5	5	5	5
5	5	5	5
5	4	4	3
5	1	1	5
4	4	5	4
5	5	5	5
5	5	4	3
5	5	5	5
4	3	4	3
5	5	4	4
5	5	5	5
4	4	4	4
5	5	5	5
5	3	1	5
5	5	5	5
4	3	3	2
4	5	4	3
5	5	1	4
4	5	5	5
5	5	5	5
4	4	4	4
4	4	4	4
5	5	4	2
1	1	1	2
5	3	3	3
5	5	3	5
5	4	4	4
5	5	4	4
5	5	5	5
5	4	4	5
5	5	3	5
5	5	5	5
3	3	2	4
4	2	5	3
4	4	4	4
4	4	3	3
4	1	3	1
4	4	4	4

4	3	5	4
5	3	5	5
5	3	4	3
4	5	5	2
5	5	5	4
4	5	5	4
5	5	5	5
3	1	2	3
5	5	5	5
4	5	4	5
4	4	5	4
4	4	5	4
4	3	5	4
5	5	5	5
4	3	4	3
5	5	5	5
5	5	5	5
5	4	4	5
5	4	4	4
5	5	4	4
5	5	5	5
5	5	3	5
5	5	5	4
3	3	3	5
5	3	5	5
5	5	4	5
5	5	5	5
3	3	3	3
5	5	5	5
4	5	5	2
5	5	5	5
5	5	5	5
3	3	3	3
2	2	4	5
2	2	4	5
4	1	3	4
4	4	4	4
3	3	2	3
3	3	3	3
2	2	1	2
4	3	2	3
4	2	4	3
5	5	4	4

5	5	3	4
5	5	2	3
5	5	5	5
5	4	3	5
3	3	3	3
2	3	5	4
2	2	1	4
4	4	3	4
5	5	5	5
5	5	5	5
5	5	5	5
	4	5	4
5	5	5	5
5	5	3	5
5	5	5	5
3	3	4	4
4	4	3	2
4	5	4	4
4	3	5	5
5	5	5	5
3	4	3	3

4	4	5	5
4	5	4	4
5	5	5	5
4	2	2	3
5	2	5	5
3	4	5	3
5	5	5	5
5	5	5	5
4	4	4	4
4	4	5	3
4	3	4	4
2	3	3	4
5	5	5	5
5	5	5	5
1	1	1	3
4	3	5	5
5	4	5	4
5	5	5	5
5	5	4	4
5	3	4	1
3	3	4	3
4	4	2	3
4	4	4	4
3	3	3	3
5	3	3	4
5	5	5	5
5	5	5	5
5	4	4	3
5	1	1	5
4	4	5	4
5	5	5	5
5	5	4	3
5	5	5	5
4	3	4	3
5	5	4	4
5	5	5	5
4	4	4	4
5	5	5	5
5	3	1	5
5	5	5	5
4	3	3	2
4	5	4	3
5	5	1	4
4	5	5	5

5	5	5	5
4	4	4	4
4	4	4	4
5	5	4	2
1	1	1	2
5	3	3	3
5	5	3	5
5	4	4	4
5	5	4	4
5	5	5	5
5	4	4	5
5	5	3	5
5	5	5	5
3	3	2	4
4	2	5	3
4	4	4	4
4	4	3	3
4	1	3	1
4	4	4	4
4	3	5	4
5	3	5	5
5	3	4	3
4	5	5	2
5	5	5	4
4	5	5	4
5	5	5	5
3	1	2	3
5	5	5	5
4	5	4	5
4	4	5	4

3	4	5	3
5	5	5	5
5	5	5	5
5	5	5	5
5	5	5	5
	4	5	4
5	5	5	5
5	5	3	5
5	5	5	5
3	3	4	4
4	4	3	2
5	5	5	4
4	5	5	4

healthlitracy27	healthlitracy28	healthlitracy29.	healthlitracy30
3	4	3	4
5	5	5	5
3	4	4	3
1	3	4	2
5	5	4	2
5	4	5	5
1	4	5	2
1	4	5	2
1	1	2	1
4	5	5	4
3	4	5	3
3	4	5	5
3	5	5	5
1	3	5	4
2	3	3	1
2	5	5	2
4	3	4	3
3	4	4	4
3	5	5	5
2	3	3	5
4	4	4	4
4	4	4	4
5	5	5	5
2	5	5	5
5	5	5	5
5	5	5	5
4	4	5	5
5	5	5	5
5	4	4	4
1	2	3	3
3	4	4	4
2	4	3	4
5	5	5	4
5	5	5	5
5	2	5	2
5	5	4	3
4	4	4	4
1	3	3	3
3	3	4	4
1	2	4	2
3	2	4	4

1	2	3	3
3	4	4	4
2	3	5	5
1	5	5	5
4	5	5	3
1	5	5	4
3	4	5	2
1	3	5	1
4	4	5	3
3	5	5	5
4	4	4	4
3	4	5	5
3	4	3	3
4	4	4	4
2	2	4	4
2	3	4	3
5	5	5	5
1	5	3	5
5	5	5	5
2	2	4	2
3	3	4	5
1	5	5	3
5	5	5	5
5	5	5	5
1	5	5	4
1	4	4	2
1	1	1	1
1	3	2	3
4	3	3	5
1	5	5	5
4	4	4	4
3	4	4	4
3	4	4	5
3	4	4	5
2	5	5	3
3	5	5	4
3	3	4	3
1	3	3	3
4	4	4	4
2	4	4	3
1	1	4	1
4	4	4	5

4	3	4	3
2	3	2	2
3	4	4	4
2	2	1	1
3	4	5	4
5	4	4	4
5	5	5	5
1	5	5	2
5	5	5	5
5	5	5	4
4	4	4	5
4	4	4	5
3	3	3	3
2	5	4	2
1	2	3	1
5	5	5	4
5	5	5	5
3	4	5	5
4	4	4	4
3	4	4	4
5	5	5	5
2	4	4	5
1	3	5	4
2		4	3
5	5	5	5
5	5	4	5
5	5	5	5
3	3	3	3
5	5	5	5
1	3	5	4
5	5	5	5
5	4	5	5
3	3	3	3
5	5	5	5
5	5	5	5
2	3	4	2
3	5	4	4
2	4	3	3
3	2	2	4
1	2	2	2
1	3	3	4
2	3	3	4
2	4	3	3

3	5	4	2
1	3	4	4
5	5	5	5
4	3	5	5
3	3	3	2
3	4	5	3
1	3	5	1
3	4	4	5
5	3	5	1
4	5	5	4
5	5	5	5
5	5	5	
4	5	5	5
2	5	5	5
3	5	4	3
5	5	5	5
2	4	5	1
5	4	4	4
3	5	5	5
5	5	5	4
4	3	4	4

4	4	4	4
4	4	4	4
5	5	5	5
2	5	5	5
5	5	5	5
5	5	5	5
4	4	5	5
5	5	5	5
5	4	4	4
1	2	3	3
3	4	4	4
2	4	3	4
5	5	5	4
5	5	5	5
5	2	5	2
5	5	4	3
4	4	4	4
1	3	3	3
3	3	4	4
1	2	4	2
3	2	4	4
1	2	3	3
3	4	4	4
2	3	5	5
1	5	5	5
4	5	5	3
1	5	5	4
3	4	5	2
1	3	5	1
4	4	5	3
3	5	5	5
4	4	4	4
3	4	5	5
3	4	3	3
4	4	4	4
2	2	4	4
2	3	4	3
5	5	5	5
1	5	3	5
5	5	5	5
2	2	4	2
3	3	4	5
1	5	5	3
5	5	5	5

5	5	5	5
1	5	5	4
1	4	4	2
1	1	1	1
1	3	2	3
4	3	3	5
1	5	5	5
4	4	4	4
3	4	4	4
3	4	4	5
3	4	4	5
2	5	5	3
3	5	5	4
3	3	4	3
1	3	3	3
4	4	4	4
2	4	4	3
1	1	4	1
4	4	4	5
4	3	4	3
2	3	2	2
3	4	4	4
2	2	1	1
3	4	5	4
5	4	4	4
5	5	5	5
1	5	5	2
5	5	5	5
5	5	5	4
4	4	4	5

5	5	5	5
4	4	5	5
5	3	5	1
4	5	5	4
5	5	5	5
5	5	5	
4	5	5	5
2	5	5	5
3	5	4	3
5	5	5	5
2	4	5	1
3	4	5	4
5	4	4	4

healthlitracy31	healthlitracy32	healthlitracy33	coping1
5	5	5	4
5	5	5	5
2	4	4	4
2	4	2	5
4	5	5	5
5	5	5	5
4	3	4	2
4	3	4	2
2	5	2	4
4	5	4	2
5	5	3	4
3	5	4	3
2	5	2	5
1	5	3	2
1	5	2	4
3	5	3	
3	4	3	4
2	5	3	4
5	4	5	3
4	2	5	2
5	5	5	5
3	5	4	3
5	5	5	2
5	5	5	5
5	1	1	5
3	3	3	5
5	5	5	4
5	5	5	5
5	4	4	4
3	5	2	3
3	5	4	
5	5	5	5
4	2	4	4
5	5	5	3
2	5	2	5
3	5	5	4
4	2	3	5
3	5	3	5
5	5	4	4
1	1	2	3
2	5	1	2

3	4	2	3
4	5	4	4
4	5	5	2
5	5	5	1
1	1	1	5
4	5	5	5
4	3	2	4
1	3	3	
3	4	4	3
5	5	3	3
4	3	3	4
5	5	5	4
4	4	4	4
4	5	3	4
3	5	4	3
3	4	4	3
5	5	5	4
5	5	4	5
5	5	5	5
4	2	2	4
4	5	3	4
5	3	5	5
5	5	5	5
5	5	5	3
4	5	4	3
4	5	3	4
1	1	1	2
1		2	4
4	4	3	3
4	5	3	4
4	5	5	3
4	4	4	3
5	5	4	4
5	5	5	4
5	5	5	3
5	4	5	4
3	5	2	3
2	4	2	4
4	1	4	5
4	4	4	4
1	2	1	1
4	5	4	4

3	5	4	2
2	3	2	4
4	5	3	4
1	4	2	1
3	4	4	3
3	4	4	4
5	5	5	4
3	5	5	3
4	5	5	5
4	4	4	5
4	3	3	3
4	5	4	4
3	4	4	3
3	5	5	5
1	4	1	2
4	5	5	4
4	5	5	4
4	4	4	
4	5	5	3
4	4	4	3
5	5	5	4
3	5	5	4
5	4	5	1
3	5	3	5
5	5	5	5
5	4	5	5
5	5	5	1
3	3	3	3
5	5	5	
4	5	2	4
5	5	5	5
4	5	5	4
3	3	3	3
5	5	5	5
5	5	5	5
4	3	4	4
4	5	4	4
3	4	3	3
3	2	4	4
2	2	2	1
2	3	3	
2	4	2	2
3	5	5	2

3	5	2	5
1	5	1	3
5	5	5	5
5	5	3	1
3	4	4	3
3	3	3	3
1	5	1	1
4	4	4	4
5	4	2	2
5	4	5	3
5	5	5	5
3	5	5	4
5	4	4	4
4	5	5	5
4	3	3	3
4	5	4	4
2	3	2	5
4	3	3	4
4	5	5	5
5	5	5	1
4	4	3	4

5	5	5	5
3	5	4	3
5	5	5	2
5	5	5	5
5	1	1	5
3	3	3	5
5	5	5	4
5	5	5	5
5	4	4	4
3	5	2	3
3	5	4	
5	5	5	5
4	2	4	4
5	5	5	3
2	5	2	5
3	5	5	4
4	2	3	5
3	5	3	5
5	5	4	4
1	1	2	3
2	5	1	2
3	4	2	3
4	5	4	4
4	5	5	2
5	5	5	1
1	1	1	5
4	5	5	5
4	3	2	4
1	3	3	
3	4	4	3
5	5	3	3
4	3	3	4
5	5	5	4
4	4	4	4
4	5	3	4
3	5	4	3
3	4	4	3
5	5	5	4
5	5	4	5
5	5	5	5
4	2	2	4
4	5	3	4
5	3	5	5
5	5	5	5

5	5	5	3
4	5	4	3
4	5	3	4
1	1	1	2
1		2	4
4	4	3	3
4	5	3	4
4	5	5	3
4	4	4	3
5	5	4	4
5	5	5	4
5	5	5	3
5	4	5	4
3	5	2	3
2	4	2	4
4	1	4	5
4	4	4	4
1	2	1	1
4	5	4	4
3	5	4	2
2	3	2	4
4	5	3	4
1	4	2	1
3	4	4	3
3	4	4	4
5	5	5	4
3	5	5	3
4	5	5	5
4	4	4	5
4	3	3	3

3	3	3	5
5	5	5	4
5	4	2	2
5	4	5	3
5	5	5	5
3	5	5	4
5	4	4	4
4	5	5	5
4	3	3	3
4	5	4	4
2	3	2	5
3	4	4	3
3	4	4	4

coping2	coping3	coping4	coping5
3	3	3	2
4	3	4	4
2	2	3	2
4	4	4	4
5	2	5	5
1	1	5	1
4	4	4	4
4	4	4	4
5	4	3	3
2	1	5	2
3	1	2	1
1	1	3	4
5	2	4	4
3	3	3	1
4	2	3	4
2	3	4	3
4	4	3	2
2	1	1	1
3	2	3	4
5	3	4	3
2	3	3	4
2	3	2	2
5	4	5	5
1	5	1	5
2	1	4	2
4	2	4	2
2	2	4	3
3	4	4	4
5	3	3	4
2	2	3	1
2	1	2	3
1	1	1	1
2	3	3	3
2	1	2	1
5	4	5	5
5	1	4	3
3	3	4	4
4	2	4	3
4	4	5	4

3	5	3	3
3	3	3	3
3	2	4	5
3	1	5	5
1	1	1	1
3	1	5	1
3		4	4
1	1	1	1
5	3	5	3
2	2	4	2
2	1	3	1
5	4	4	4
2	2	3	2
3	4	4	4
2	2	4	2
1	2	5	4
1	2	5	2
5	5	5	5
4	4	4	4
1	1	3	3
5	5	5	5
5	5	5	5
4	2	4	2
4	3	3	3
2	1	4	3
4	5	5	4
4	3	4	3
3	4	5	3
2	1	3	1
4	3	5	4
2	2	3	2
4	3	3	2
3	2	4	1
2	1	5	5
5	4	4	5
5	4	4	3
2	3	4	3
4	4	4	4
4	1	3	2
2	1	5	3
4	4	4	4

3	5	5	4
2	2	5	3
2	3	5	5
3	5	1	1
4	2	4	3
4	4	5	5
3	1	4	1
1	1	5	2
2	1	5	1
4	2	5	5
3	1	2	2
4	2	5	3
2	3	3	2
1	3	5	5
5	3	1	1
4	2	4	2
1	1		1
4	3	5	4
2	2	3	2
1	2	5	1
3	3	4	2
2	3	3	2
2	1	4	3
5	1	5	5
4	2	3	3
1	1	3	1
3	2	3	3
5		5	2
2	1	4	1
2	1	5	1
4	1	1	4
3	3	3	3
2	2	5	3
2	2	5	3
3	2	4	2
3	1	3	3
1	1	4	3
3	3	4	3
5	5	5	5
3	1	5	1
4	4	4	2
5	1	3	2

3	4	4	5
4	5	5	4
5	1	5	1
1	1	2	1
2	2	3	3
3	4	5	3
1	4	3	4
5	1	4	4
5	2	3	5
3	1	3	1
4	1	5	5
4	4	4	4
5	1	5	5
3	4	4	3
3	2	4	3
2	2	3	2
2	2	2	2
4		5	2
3	3	5	4
1	1	3	1
3	4	4	4

5	3	4	3
2	3	3	4
2	3	2	2
5	4	5	5
1	5	1	5
2	1	4	2
4	2	4	2
2	2	4	3
3	4	4	4
5	3	3	4
2	2	3	1
2	1	2	3
1	1	1	1
2	3	3	3
2	1	2	1
5	4	5	5
5	1	4	3
3	3	4	4
4	2	4	3
4	4	5	4
3	5	3	3
3	3	3	3
3	2	4	5
3	1	5	5
1	1	1	1
3	1	5	1
3		4	4
1	1	1	1
5	3	5	3
2	2	4	2
2	1	3	1
5	4	4	4
2	2	3	2
3	4	4	4
2	2	4	2
1	2	5	4
1	2	5	2
5	5	5	5
4	4	4	4
1	1	3	3
5	5	5	5
5	5	5	5

4	2	4	2
4	3	3	3
2	1	4	3
4	5	5	4
4	3	4	3
3	4	5	3
2	1	3	1
4	3	5	4
2	2	3	2
4	3	3	2
3	2	4	1
2	1	5	5
5	4	4	5
5	4	4	3
2	3	4	3
4	4	4	4
4	1	3	2
2	1	5	3
4	4	4	4
3	5	5	4
2	2	5	3
2	3	5	5
3	5	1	1
4	2	4	3
4	4	5	5
3	1	4	1
1	1	5	2
2	1	5	1
4	2	5	5
3	1	2	2

2	1	4	2
4	2	4	2
5	2	3	5
3	1	3	1
4	1	5	5
4	4	4	4
5	1	5	5
3	4	4	3
3	2	4	3
2	2	3	2
2	2	2	2
4	2	4	3
4	4	5	5

coping6	coping7.	coping8	coping9
3	4	3	5
3	5	4	5
3	4	2	4
2	3	4	3
5	5	5	5
5	5	5	5
4	4	3	2
4	4	3	2
3	3	5	2
2	5	4	5
3	5	3	4
4	5	4	3
2	5	3	4
1	3	3	2
2	4	2	4
3	3	2	3
3	5	5	3
2	2	2	3
5	5	5	5
3	3	3	3
3	3	3	4
3	5	4	1
5	5	4	4
5	5	5	4
5	5	2	5
4	4	2	4
2	5	4	4
4	4	4	4
3	4	4	4
3	4	5	4
1	3	1	3
1	5	3	5
3	5	5	5
3	5	2	4
5	5	4	5
4	4	3	5
3	4	3	2
3	4	4	4
4	4	5	4

3	4	5	4
3	3	4	4
3	5	5	5
3	3	5	5
3	5	3	5
1	2	1	5
3	5	5	4
1	2	1	2
3	5	5	5
2	2	2	4
1	5	1	5
5	4	3	4
1	4	3	4
4	5	3	2
3	4	3	4
3	4	4	4
3	3	5	5
5	5	5	5
4	5	4	4
1	4	3	4
5	5	5	5
5	5	5	5
4	2	2	4
5	4	3	3
4	5	5	5
4	4	4	1
3	4	4	3
4	4	3	4
3	3	3	4
4	4	4	3
3	4	3	5
2	5	4	5
2	4	3	4
3	5	2	3
3	4	4	4
3	2	2	3
3	4	3	3
4	4	4	3
4	4	3	5
	3	2	5
4	4	4	4

3	3	3	3
3	4	4	3
3	4	5	3
2	3	3	3
1	5	5	5
5	5	5	5
3	4	4	4
5	5	5	4
2	3	4	5
2	3	1	4
2	4	4	4
3	3	2	4
3	3	3	3
5	5	4	5
1	1		
2	1	2	2
2	5	3	5
4	4	4	3
3	4	3	5
4	1	5	5
3	4	4	4
3	3	4	2
3	3	3	3
5	5	5	5
3	3	1	4
3	3	3	3
3	3	3	3
5	3	5	5
2	3	3	1
4	3	4	5
1	5	2	4
3	3	3	3
2	4	2	4
2	4	2	4
3	4	4	4
4	5	3	4
4	3	2	4
3	5	3	4
2	5	5	2
3	3	3	1
4	4	5	3
5	4	4	4

2	4	4	5
3	5	5	5
1	2	1	5
3	4	3	4
4	4	3	4
5	4	4	5
5	5	5	5
4	5	3	5
3	5	2	2
2	4	3	3
5	5	5	5
4	3	2	4
4	5	4	5
4	5	3	2
2	3	3	4
4	4	4	4
3	5	4	5
4	4	3	4
3	4	4	5
1	5	5	4
4	4	3	4

3	3	3	3
3	3	3	4
3	5	4	1
5	5	4	4
5	5	5	4
5	5	2	5
4	4	2	4
2	5	4	4
4	4	4	4
3	4	4	4
3	4	5	4
1	3	1	3
1	5	3	5
3	5	5	5
3	5	2	4
5	5	4	5
4	4	3	5
3	4	3	2
3	4	4	4
4	4	5	4
3	4	5	4
3	3	4	4
3	5	5	5
3	3	5	5
3	5	3	5
1	2	1	5
3	5	5	4
1	2	1	2
3	5	5	5
2	2	2	4
1	5	1	5
5	4	3	4
1	4	3	4
4	5	3	2
3	4	3	4
3	4	4	4
3	3	5	5
5	5	5	5
4	5	4	4
1	4	3	4
5	5	5	5
5	5	5	5

4	2	2	4
5	4	3	3
4	5	5	5
4	4	4	1
3	4	4	3
4	4	3	4
3	3	3	4
4	4	4	3
3	4	3	5
2	5	4	5
2	4	3	4
3	5	2	3
3	4	4	4
3	2	2	3
3	4	3	3
4	4	4	3
4	4	3	5
	3	2	5
4	4	4	4
3	3	3	3
3	4	4	3
3	4	5	3
2	3	3	3
1	5	5	5
5	5	5	5
3	4	4	4
5	5	5	4
2	3	4	5
2	3	1	4
2	4	4	4

5	5	2	5
4	4	2	4
3	5	2	2
2	4	3	3
5	5	5	5
4	3	2	4
4	5	4	5
4	5	3	2
2	3	3	4
4	4	4	4
3	5	4	5
1	5	5	5
5	5	5	5

coping10	coping11	coping12	coping13
5	3	3	4
4	4	5	4
4	4	3	4
3	2	4	4
5	5	5	5
5	5	5	5
2	4	3	1
2	4	3	1
2	2	2	3
5	4	4	1
4	4	3	5
3	4	4	4
4	4	5	5
2	5	4	2
4	4	3	5
4	3	3	3
3	5	5	5
3	2	2	5
5	5	4	5
3	4	3	3
4	3	4	4
1	1	3	1
4	4	5	5
4	5	1	1
5	4	5	5
4	2	3	4
4	4	4	5
4	4	4	4
4	4	4	5
4	4	4	5
3	4	1	5
5	5	4	5
5	3	1	5
4	5	5	5
5	4	4	4
5	1	4	5
2	5	4	4
4	4	4	4
4	4	5	4

4	3	4	4
4	4	4	5
5	4	4	5
5	5	5	5
5	5	3	5
5	5	5	5
4	3	4	5

2	2	3	5
5	5	5	5
4	4	4	4
5	1	2	5
5	4	4	4
4	3	4	5
2	2	4	3
4	3	3	4
4	5	5	3
5	3	3	5
5	5	5	5
4	4	4	4
4	4	4	4
5	5	5	5
5	5	5	5
4	3	5	4

3	3	3	3
5	3	5	5
1	1	1	1
3	3	3	4
3	4	3	3

4	4	3	5
3	3	5	4
5	4	4	4
5	4	5	3
4	3	3	3
3	4	5	4
4	4	4	4
3	2	2	3
3	4	4	4
3	3	3	3
4	3	3	5
5	5	1	5
5	4	4	4

4	4	5	2
3	4	4	2
3	3	4	5
3	2	2	2
5	4	5	3
5	5	5	5
3	4	5	4
4	3	5	5
5	4	4	
4	4	5	4
4	3	4	5
4	4	4	4
3	3	3	3
5	5	5	5
2	1	2	1
2	2	1	1
5	5	5	5
3	3	5	4
5	4	4	4
5	5	5	4
4	4	3	3
2	4	3	2
3	5	3	5
5	1	5	5
4	4	4	5
3	3	3	3
3	3	3	3
4	4	4	5
1	3	4	4
5	5	5	5
4	3	5	5
3	3	3	3
4	5	4	5
4	5	4	5
4	4	4	4
4	4	4	4
4	2	3	4
4	4	3	4
2	2	5	5
2	4	4	3
3	4	4	2
4	5	4	5

5	3	3	4
5	5	5	5
5	4	5	5
4	1	2	5
4	4	4	4
5	4	5	5
5	5	5	5
5	5	5	5
2	4	5	5
3	3	3	5
5	5	5	5
4	3	3	3
5	4	5	5
2	4	5	5
4	4	4	4
4	4	4	3
5	2	3	5
4	2	4	4
5	5	5	1
4	2	4	5
3	5	4	4

3	4	3	3
4	3	4	4
1	1	3	1
4	4	5	5
4	5	1	1
5	4	5	5
4	2	3	4
4	4	4	5
4	4	4	4
4	4	4	5

4	4	4	5
3	4	1	5
5	5	4	5
5	3	1	5
4	5	5	5
5	4	4	4
5	1	4	5
2	5	4	4
4	4	4	4
4	4	5	4
4	3	4	4
4	4	4	5
5	4	4	5
5	5	5	5
5	5	3	5
5	5	5	5
4	3	4	5

2	2	3	5
5	5	5	5
4	4	4	4
5	1	2	5
5	4	4	4
4	3	4	5
2	2	4	3
4	3	3	4
4	5	5	3
5	3	3	5
5	5	5	5
4	4	4	4
4	4	4	4
5	5	5	5
5	5	5	5

4	3	5	4
3	3	3	3
5	3	5	5
1	1	1	1
3	3	3	4
3	4	3	3
4	4	3	5
3	3	5	4
5	4	4	4
5	4	5	3
4	3	3	3
3	4	5	4
4	4	4	4
3	2	2	3
3	4	4	4
3	3	3	3
4	3	3	5
5	5	1	5
5	4	4	4
4	4	5	2
3	4	4	2
3	3	4	5
3	2	2	2
5	4	5	3
5	5	5	5
3	4	5	4
4	3	5	5
5	4	4	
4	4	5	4
4	3	4	5

5	4	5	5
4	2	3	4
2	4	5	5
3	3	3	5
5	5	5	5
4	3	3	3
5	4	5	5
2	4	5	5
4	4	4	4
4	4	4	3
5	2	3	5
5	4	5	3
5	5	5	5

coping14.	coping15	coping16	coping17
5	1	1	3
3	2	1	1
4	3	3	4
4	5	2	2
5	5	5	4
5	1	1	1
1	4	4	2
1	4	4	2
1	3	4	3
4	1	1	2
2	4	2	3
3	2	1	1
2	1	2	3
5	3	1	2
5	2	2	2
4	2	4	3
4	5	5	2
3	1	1	2
5	1	1	5
3	3	3	3
3	2	2	4
1	1	1	1
4	5	3	4
5	1	1	1
2	1	1	1
3	1	1	3
5	1	1	3
4	3	3	4
5	4	2	4
4	1	2	2
4	2	1	3
5	1	1	1
3	2	1	5
4	1	1	3
3	4	4	4
5	5	3	5
4	3	1	2
4	4	2	4
2	4	4	2

4	3	3	1
4	3	3	3
5	4	4	5
3	1	1	1
4	1	1	1
3	1	1	5
2	2	1	1
3	3	3	2
3	3	3	3
4	2	2	2
3	1	1	2
4	4	4	4
4	1	1	4
2	4	3	4
3	1	1	2
2	5	3	1
2	1	1	5
5	5	5	5
2	1	1	2
3	1	1	1
1	1	1	5
5	5	5	5
3	2	1	1
4	2	3	3
5	1	1	2
2	3	3	3
3	3	3	4
4	3	4	3
1	1	1	1
3	2	2	3
3	2	2	2
5	4	2	5
3	1	1	2
2	1	1	2
4	4	4	4
2	3	4	3
4	1	2	3
4	5	1	2
5	1	2	2
5	3	3	2
4	3	3	3

1	1	1	5
4	2	1	3
4	3	2	2
4	5	3	3
2	2	1	5
3	3	3	4
3	2	1	3
1	3	1	1
5	1	1	1
1	1	1	4
2	1	1	2
3	4	2	4
3	3	3	3
3	1	1	5
5	5	5	5
2	1	1	1
5	1	1	3
3	2	2	3
3	2	2	2
1	1	1	5
4	3	2	2
2	1	1	2
5	1	1	1
5	1	1	1
4	3	2	4
3	1	1	3
1	1	3	1
2	1	1	4
5	1	1	4
5	1	1	5
1	1	1	1
3	3	3	3
5	2	2	2
5	2	2	2
2	4	4	4
3	1	1	2
3	2	1	2
3	4	3	3
5	4	5	4
4	1	1	5
2	3	3	4
5	3	2	2

3	1	1	3
5	1	1	1
5	1	1	4
4	1	1	2
3	1	1	2
4	4	2	5
5	4	3	3
5	1	3	2
3	1	1	4
3	2	1	2
5	5	5	5
3	3	3	3
1	1	1	4
4	4	2	2
2	1	1	1
3	1	1	2
5	1	1	3
4	2	2	4
4	1	2	1
4	4	2	1
5	5	5	3

3	3	3	3
3	2	2	4
1	1	1	1
4	5	3	4
5	1	1	1
2	1	1	1
3	1	1	3
5	1	1	3
4	3	3	4
5	4	2	4
4	1	2	2
4	2	1	3
5	1	1	1
3	2	1	5
4	1	1	3
3	4	4	4
5	5	3	5
4	3	1	2
4	4	2	4
2	4	4	2
4	3	3	1
4	3	3	3
5	4	4	5
3	1	1	1
4	1	1	1
3	1	1	5
2	2	1	1
3	3	3	2
3	3	3	3
4	2	2	2
3	1	1	2
4	4	4	4
4	1	1	4
2	4	3	4
3	1	1	2
2	5	3	1
2	1	1	5
5	5	5	5
2	1	1	2
3	1	1	1
1	1	1	5
5	5	5	5

3	2	1	1
4	2	3	3
5	1	1	2
2	3	3	3
3	3	3	4
4	3	4	3
1	1	1	1
3	2	2	3
3	2	2	2
5	4	2	5
3	1	1	2
2	1	1	2
4	4	4	4
2	3	4	3
4	1	2	3
4	5	1	2
5	1	2	2
5	3	3	2
4	3	3	3
1	1	1	5
4	2	1	3
4	3	2	2
4	5	3	3
2	2	1	5
3	3	3	4
3	2	1	3
1	3	1	1
5	1	1	1
1	1	1	4
2	1	1	2

2	1	1	1
3	1	1	3
3	1	1	4
3	2	1	2
5	5	5	5
3	3	3	3
1	1	1	4
4	4	2	2
2	1	1	1
3	1	1	2
5	1	1	3
2	2	1	5
3	3	3	4

coping18.	coping19	coping20	coping21
5	5	5	5
4	5	4	5
4	4	4	4
2	2	2	3
5	5	3	4
1	5	5	5
1	5		5
1	5		5
4	3	4	3
3	5	5	2
2	4	4	4
5	4	4	4
3	4	4	4
1	2	1	1
4	4	4	4
4	4	4	3
4	5	5	4
3	3	3	3
3	3	4	2
3	4	4	3
4	2	2	4
5	2	1	2
3	1	1	4
1	1	1	1
5	5	5	5
5	4	4	3
4	5	5	5
4	4	4	4
4	3	3	3
5	5	4	4
4	4	4	1
5	5	5	5
4	3	2	1
5	5	5	5
5	5	5	5
1	4	4	1
3	4	4	4
1	3	5	5
3	4	4	4

1	2	2	3
3	4	4	4
5	5	5	5
3	2	2	1
5	5	5	1
5	5	5	5
4	4	4	5
5	5	5	5
4	4	4	4
4	2	2	2
3	4	4	2
4	4	4	4
4	3	3	4
3	3	3	3
3	4	4	3
5	1	1	4
4	5	5	4
5	5	5	5
3	5	5	4
4	4	4	3
5	5	5	5
5	5	5	5
5	3	3	4
3	3	3	3
4	5	5	3
2	3	3	3
2	2	2	2
3	3	3	4
3	4	4	4
5	5	5	4
3	3	3	3
5	5	5	5
4	2	3	2
2	2	2	2
4	4	4	4
3	3	3	3
5	5	5	3
3	3	3	3
5	5	5	3
1	3	3	3
3	3	3	3

1	3	4	3
3	4	4	1
4	5	5	3
1	2	3	1
3	3	5	3
4	4	4	4
4	4	4	3
5	5	5	3
4	5	5	5
4	4	4	4
5	5	4	3
5	5	5	5
3	3	3	3
5	5	5	5
1	3	3	2
3	5	5	5
5	5	5	5
5	5	5	4
3	3	3	3
1	5	5	3
4	3	4	4
5	3	3	3
5	4	4	4
5	5	5	5
4	5	5	4
5	5	5	3
3	3	3	3
4	5	5	5
1	5	5	3
4	5	5	4
5	5	5	5
3	3	3	3
4	5	5	2
4	5	5	2
4	3	3	4
3	5	5	5
3	3	3	2
3	4	3	4
5	1	1	1
4	4	4	4
4	2	2	3
1	3	3	5

4	4	4	5
3	3	3	4
5	5	5	5
2	5	5	5
3	4	4	4
5	5	5	3
4	4	4	4
3	5	5	5
3	5	5	4
5	4	4	3
4	5	5	5
3	4	3	3
3	2	2	2
3	4	4	3
4	4	4	3
4	4	4	4
4	4	4	2
4	5	4	4
4	5	5	4
1	1	3	3
3	4	3	5

3	4	4	3
4	2	2	4
5	2	1	2
3	1	1	4
1	1	1	1
5	5	5	5
5	4	4	3
4	5	5	5
4	4	4	4
4	3	3	3
5	5	4	4
4	4	4	1
5	5	5	5
4	3	2	1
5	5	5	5
5	5	5	5
1	4	4	1
3	4	4	4
1	3	5	5
3	4	4	4
1	2	2	3
3	4	4	4
5	5	5	5
3	2	2	1
5	5	5	1
5	5	5	5
4	4	4	5
5	5	5	5
4	4	4	4
4	2	2	2
3	4	4	2
4	4	4	4
4	3	3	4
3	3	3	3
3	4	4	3
5	1	1	4
4	5	5	4
5	5	5	5
3	5	5	4
4	4	4	3
5	5	5	5
5	5	5	5

5	3	3	4
3	3	3	3
4	5	5	3
2	3	3	3
2	2	2	2
3	3	3	4
3	4	4	4
5	5	5	4
3	3	3	3
5	5	5	5
4	2	3	2
2	2	2	2
4	4	4	4
3	3	3	3
5	5	5	3
3	3	3	3
5	5	5	3
1	3	3	3
3	3	3	3
1	3	4	3
3	4	4	1
4	5	5	3
1	2	3	1
3	3	5	3
4	4	4	4
4	4	4	3
5	5	5	3
4	5	5	5
4	4	4	4
5	5	4	3

5	5	5	5
5	4	4	3
3	5	5	4
5	4	4	3
4	5	5	5
3	4	3	3
3	2	2	2
3	4	4	3
4	4	4	3
4	4	4	4
4	4	4	2
3	3	5	3
4	4	4	4

mental health1	mental health2	mental health3	mental health4
1	1	1	1
2	1	1	1
2	2	1	1
2	1	2	1
1	1	3	1
4	1	1	1
2	4	4	1
2	4	4	1
4	4	4	2
1	1	1	1
1	1	1	1
2	1	1	1
2	2	1	1
2	4	3	2
2	1	1	1
3	3	2	2
4	4	1	4
1	2	1	1
3	3	3	2
3	2	1	1
3	2	1	1
1	1	1	1
3	4	1	1
1	1	1	1
2	1	1	1
1	3	1	3
2	1	1	1
2	2	2	2
3	1	1	1
4	3	1	1
1	1	1	1
1	1	1	1
3	3	1	1
2	1	1	1
1	1	1	1
1	1	1	2
3	2	3	2
1	1	1	1
3	1	2	1

4	4	3	3
2	1	2	1
3	1	1	1
2	2	1	1
2	1	1	1
1	1	1	1
2	2	1	1

1	2	2	1
3	2	1	1
2	1	1	1
1	3	1	1
1	2	1	2
2	1	1	1
1	1	3	4
1	2	2	1
4	1	1	1
1	1	1	3
2	1	1	1
3	2	3	1
2	1	1	1
1	1	1	1
1	3	1	1
2	1	1	1

3	1	1	1
2	1	1	2
4	4	4	3
2	1	1	1
2	3	2	2

1	1	1	1
2	2	2	1
2	2	1	1
1	1	1	1
1	1	1	1
1	1	1	1
3	1	4	1
2	2	3	2
2	2	1	2
4	3	3	2
3	1	1	1
4	2	2	1
3	2	2	2

4	3	4	2
1	2	1	1
2	1	1	1
4	2	4	2
2	1	1	1
3	2	2	1
3	2	1	1
2	3	1	3
1	1	1	1
2	1	1	1
2	3	1	1
1	1	1	1
2	2	2	2
1		1	1
4	1	1	1
2	1	1	1
1	1	1	1
2	2	2	1
2	2	1	1
2	1	1	1
3	1	2	2
4	3	3	1
1	1	1	1
1	1	1	1
3	3	4	1
1	1	1	1
1	1	1	1
1	1	1	1
2	1		1
3	1	1	1
1	1	1	1
2	2	2	2
2	1	1	2
2	1	1	2
2	2	2	2
2	1	1	1
1	1	1	1
3	3	2	2
4	1	4	3
2	1	1	2
3	1	1	2
4	2	3	2

1	1	1	1
4	4	2	3
1	1	1	1
4	3	2	2
2	1	2	1
2	2	2	2
4	3	2	2
1	3	1	1
4	4	1	2
1	1	1	1
1	1	1	1
2	1	1	1
1	1	1	1
2	1	1	1
1	1	1	1
3	1	2	2
2	3	1	1
3	1	1	2
4	2	1	1
1	1	1	1
3	3	3	3

3	2	1	1
3	2	1	1
1	1	1	1
3	4	1	1
1	1	1	1
2	1	1	1
1	3	1	3
2	1	1	1
2	2	2	2
3	1	1	1
4	3	1	1
1	1	1	1
1	1	1	1
3	3	1	1
2	1	1	1
1	1	1	1
1	1	1	2
3	2	3	2
1	1	1	1
3	1	2	1
4	4	3	3
2	1	2	1
3	1	1	1
2	2	1	1
2	1	1	1
1	1	1	1
2	2	1	1
1	2	2	1
3	2	1	1
2	1	1	1
1	3	1	1
1	2	1	2
2	1	1	1
1	1	3	4
1	2	2	1
4	1	1	1
1	1	1	3
2	1	1	1
3	2	3	1
2	1	1	1
1	1	1	1
1	3	1	1

2	1	1	1
3	1	1	1
2	1	1	2
4	4	4	3
2	1	1	1
2	3	2	2
1	1	1	1
2	2	2	1
2	2	1	1
1	1	1	1
1	1	1	1
1	1	1	1
3	1	4	1
2	2	3	2
2	2	1	2
4	3	3	2
3	1	1	1
4	2	2	1
3	2	2	2
4	3	4	2
1	2	1	1
2	1	1	1
4	2	4	2
2	1	1	1
3	2	2	1
3	2	1	1
2	3	1	3
1	1	1	1
2	1	1	1
2	3	1	1

2	1	1	1
1	3	1	3
4	4	1	2
1	1	1	1
1	1	1	1
2	1	1	1
1	1	1	1
2	1	1	1
1	1	1	1
3	1	2	2
2	3	1	1
2	1	1	1
3	2	2	1

mental health5	mental health6	mental health7	mental health8
1	1	1	2
1	1	1	2
2	1	1	1
3	2	1	1
3	4	1	4
1	4	1	3
2	2	3	4
2	2	3	4
3	4	1	4
1	1	1	1
1	2	1	2
3	2	1	3
1	2	1	2
3	1	3	4
2	1	1	2
2	1	2	3
2	4	4	4
1	1	1	1
2	2	2	4
1	1	1	1
1	1	1	2
1	1	1	1
1	3	1	4
1	1	1	1
1	2	1	
2	2	2	3
2	2	1	2
2	2	2	2
2	2	1	2
2	1	1	3
1	2	1	1
1	1	1	1
2	2	1	1
2	3	1	1
1	3	1	2
3	1	1	1
3	4	1	4
1	1	1	1
3	3	2	4

4	4	3	4
1	2	1	2
2	3	2	3
2	1	1	1
1	1	1	1
1	1	1	1
1	1	1	2
1	1	1	1
2	2	1	4
1	2	1	2
1	3	3	2
2	1	1	1
2	1	1	1
4	1	1	3
2	3	1	1
1	4	1	4
3	1	1	1
2	2	1	4
1	2	1	4
1	1	1	4
1	1	1	1
1	1	1	1
1	2	1	2
1	1	1	1
1	2	2	2
4	3	4	4
3	3	3	2
1	2	2	2
1	1	1	1
2	2	1	2
2	3	1	3
1	1	1	2
2	2	2	4
1	2	1	2
2	2	1	3
3	3	3	2
2	2	2	3
4	4	1	3
3	1	1	2
4	4	3	4
2	3	2	2

4	4	2	4
2	2	1	3
2	2	1	3
2	3	2	3
1	2	1	3
2	3	2	2
2	3	1	2
3	4	4	2
1	1	1	1
1	2	1	1
2	2	1	2
1	1	2	2
3	2	1	2
2	1	3	1
4	3	3	4
1	1	1	1
2	2	1	2
2	2	1	2
2	3	1	3
1	2	1	1
3	2	3	3
1	3	2	4
1	1	1	3
1	1	1	4
1	2	1	1
1	1	1	1
1	2	1	1
1	2	1	2
1	4	1	3
1	3	1	2
1	1	1	1
2	2	2	2
1	2	1	2
1	2	1	2
2	3	2	3
1	1	1	1
1	1	1	1
3	2	3	2
4	1	1	4
3	1	1	2
2	3	2	3
4	4	4	4

1	3	1	3
4	3	4	4
1	1	1	1
1	4	2	2
1	2	1	1
2	1	2	4
4	4	2	4
2	2	3	2
2	3	2	3
1	1	1	1
1	1	1	1
1	1	1	2
1	1	1	1
2	2	1	1
1	1	1	1
1	2	3	3
3	2	1	2
3	3	2	2
1	1	1	1
1	1	1	1
2	3	3	3

1	1	1	1
1	1	1	2
1	1	1	1
1	3	1	4
1	1	1	1
1	2	1	
2	2	2	3
2	2	1	2
2	2	2	2
2	2	1	2
2	1	1	3
1	2	1	1
1	1	1	1
2	2	1	1
2	3	1	1
1	3	1	2
3	1	1	1
3	4	1	4
1	1	1	1
3	3	2	4
4	4	3	4
1	2	1	2
2	3	2	3
2	1	1	1
1	1	1	1
1	1	1	1
1	1	1	2
1	1	1	1
2	2	1	4
1	2	1	2
1	3	3	2
2	1	1	1
2	1	1	1
4	1	1	3
2	3	1	1
1	4	1	4
3	1	1	1
2	2	1	4
1	2	1	4
1	1	1	4
1	1	1	1
1	1	1	1

1	2	1	2

1	1	1	1
1	2	2	2
4	3	4	4
3	3	3	2
1	2	2	2

1	1	1	1
2	2	1	2
2	3	1	3
1	1	1	2
2	2	2	4
1	2	1	2
2	2	1	3
3	3	3	2
2	2	2	3
4	4	1	3
3	1	1	2
4	4	3	4
2	3	2	2
4	4	2	4
2	2	1	3
2	2	1	3
2	3	2	3

1	2	1	3
2	3	2	2
2	3	1	2
3	4	4	2
1	1	1	1
1	2	1	1
2	2	1	2

1	2	1	
2	2	2	3
2	3	2	3
1	1	1	1
1	1	1	1
1	1	1	2
1	1	1	1
2	2	1	1
1	1	1	1
1	2	3	3
3	2	1	2
1	2	1	3
2	3	2	2

mental health9	mental health10	mental health11	mental health12
1	3	3	1
1	2	2	1
2	1	1	1
1	3	3	2
3	2		3
1	1	1	1
4	3	3	4
4	3	3	4
4	4	4	4
1	1	1	1
1	1	2	2
1	3	4	1
2	1	1	1
3	3	2	2
3	3	2	1
3	3	2	3
4	1	3	4
1	1	1	1
3	3	3	3
1	1	1	1
2	2	2	2
1	1	1	1
3	1	1	1
1	4	4	1
2	1	1	1
2	1	1	1
2	2	2	3
2	2	2	2
4	1	3	2
1	2	2	3
1	1	1	1
1	1	1	1
1	3	1	1
1	1	1	1
2	1	1	2
1	1	1	1
4	2	3	2
1	1	1	1
4	3	3	3

3	4	4	4
2	2	2	2
4		4	4
2	4	4	4
1	1	1	1
1	1	1	1
3	3	1	2
1	1	1	1
2	1	2	2
2	2	1	2
1	1	1	1
1	1	1	1
1	1	1	2
2	4	4	3
2	2	1	2
1	1	1	1
4	1	1	1
2	1	1	1
3	3	3	2
1	1	2	1
2	1	1	1
1	1	1	1
1	1	1	2
1	1	1	1
2	1	2	1
4	4	4	4
3	2	2	2
3	2	3	3
1	2	1	1
1	1	1	2
1	1	1	1
1	1	1	2
3	2	2	1
1	1	1	1
2	3	4	2
2	2	2	2
2	2	3	3
3	3	3	2
1	2	1	2
4	1	2	2
1	2	2	2

4	4	4	4
3	2	2	1
1	2	2	1
3	4	4	3
2	1	2	2
2	2	2	2
3	2	2	3
1	1	2	1
1	1	1	1
1	1	1	2
3	1	2	1
1	1	1	1
1	2	2	2
4	1	1	2
4	4	4	4
1	1	1	1
2	1	1	1
1	1	1	2
1	1	1	1
	1	1	1
3	3	2	2
4	3	4	4
1	1	2	3
1	1	1	1
1	1	2	1
1	1	1	1
1	1	1	1
1	1	1	1
2	1	1	3
1	1	1	2
1	1	1	1
2	2	2	2
3	1	1	1
3	1	1	1
4	3	1	2
1	1	1	1
1	1	1	1
3	2	2	3
4	4	4	4
2	1	2	2
3	1	3	2
4	4	4	3

1	1	2	1
4	4	4	4
1	1	1	1
1	1	3	3
1	1	1	2
3	1	4	2
3	3	4	4
4	1	1	1
1	2	2	3
1	1	1	1
1	1	1	1
1	1	1	1
1	1	1	1
1	1	2	1
2	1	1	1
1	4	3	3
2	1	1	1
2	1	1	1
1	1	1	1
1	1	1	1
3	3	3	3

1	1	1	1
2	2	2	2
1	1	1	1
3	1	1	1
1	4	4	1
2	1	1	1
2	1	1	1
2	2	2	3
2	2	2	2
4	1	3	2
1	2	2	3
1	1	1	1
1	1	1	1
1	3	1	1
1	1	1	1
2	1	1	2
1	1	1	1
4	2	3	2
1	1	1	1
4	3	3	3
3	4	4	4
2	2	2	2
4		4	4
2	4	4	4
1	1	1	1
1	1	1	1
3	3	1	2
1	1	1	1
2	1	2	2
2	2	1	2
1	1	1	1
1	1	1	1
1	1	1	2
2	4	4	3
2	2	1	2
1	1	1	1
4	1	1	1
2	1	1	1
3	3	3	2
1	1	2	1
2	1	1	1
1	1	1	1

1	1	1	2
1	1	1	1
2	1	2	1
4	4	4	4
3	2	2	2
3	2	3	3
1	2	1	1
1	1	1	2
1	1	1	1
1	1	1	2
3	2	2	1
1	1	1	1
2	3	4	2
2	2	2	2
2	2	3	3
3	3	3	2
1	2	1	2
4	1	2	2
1	2	2	2
4	4	4	4
3	2	2	1
1	2	2	1
3	4	4	3
2	1	2	2
2	2	2	2
3	2	2	3
1	1	2	1
1	1	1	1
1	1	1	2
3	1	2	1

2	1	1	1
2	1	1	1
1	2	2	3
1	1	1	1
1	1	1	1
1	1	1	1
1	1	1	1
1	1	2	1
2	1	1	1
1	4	3	3
2	1	1	1
2	1	2	2
2	2	2	2

mental health13	mental health14	mental health15	mental health16.
1	1	1	1
2	1	1	2
1	1	1	1
3	2	1	3
3	3	3	3
1	1	1	1
4	3	3	3
4	3	3	3
4	4	4	4
1	1	1	2
2	2	1	2
2	2	1	1
1	2	1	1
3	2	2	4
1	3	1	1
3	3	3	4
4	4	4	2
1	1	1	1
4	3	3	3
1	1	1	1
2	2	1	2
1	1	1	1
1	1	1	1
4	1	1	1
1	1	1	1
2	2	1	3
2	2	1	2
2	2	2	2
2	2	1	2
1	3	1	1
1	1	1	1
1	1	1	1
1	1	1	2
1	1	1	2
2	2	1	2
1	1	1	4
2	1	3	3
1	1	1	1
3	3	2	4

4	4	4	4
2	2	2	2
3	4	2	3
4	2	1	3
1	4	1	1
1	1	1	1
1	1	1	2
1	1	1	1
2	2	1	1
1	1	1	1
2	1	1	1
1	1	2	1
1	2	1	2
4	2	2	4
3	1	1	3
1	4	1	3
1	1	1	1
1	1	1	2
3	3	1	1
1	1	1	1
1	1	1	1
1	1	1	1
1	2	1	1
1	1	1	1
2	1	1	1
4	4	4	4
2	2	2	2
	3	3	2
1	1	1	1
2	2	2	2
1	2	1	2
1	1	1	2
2	2	1	3
1	2	1	3
1	1	1	1
2	2	2	2
1	1	1	2
4	3	2	2
3	2	2	2
3	1	3	4
2	2	1	2

4	4	4	4
2	1	1	1
2	2	1	2
4	4	2	4
2	3	1	1
3	2	2	2
2	3	1	2
1	2	1	4
1	1	1	1
1	1	1	1
1	3	2	1
3	1	1	2
2	2	2	2
1	1	1	3
4	4	4	4
1	1	1	1
1	1	1	1
2	2	2	2
1	2	1	2
1	1	1	1
1	1	2	3
4	4	2	4
2	1	1	3
1	1	1	1
1	1	1	1
1	1	1	1
1	1	1	1
1	1	1	1
1	2	1	4
1	3	1	2
1	1	1	1
2	2	2	2
1	1	1	1
1	1	1	1
3	3	2	3
1	2	1	2
1	2	1	2
4	2	3	2
4	3	3	4
2	2	1	3
4	3	2	2
3	4	2	3

2	2	1	1
4	4	4	4
1	1	1	1
2	3	1	3
2	2	1	2
4	1	4	2
1	4	4	2
1	1	1	3
2	3	3	4
1	1	1	1
1	1	1	1
1	1	1	1
1	1	1	2
2	1	1	1
1	1	1	1
3	2	1	4
2	2	1	1
1	1	1	1
1	2	1	1
1	2	2	4
3	3	2	3

1	1	1	1
2	2	1	2
1	1	1	1
1	1	1	1
4	1	1	1
1	1	1	1
2	2	1	3
2	2	1	2
2	2	2	2
2	2	1	2
1	3	1	1
1	1	1	1
1	1	1	1
1	1	1	2
1	1	1	2
2	2	1	2
1	1	1	4
2	1	3	3
1	1	1	1
3	3	2	4
4	4	4	4
2	2	2	2
3	4	2	3
4	2	1	3
1	4	1	1
1	1	1	1
1	1	1	2
1	1	1	1
2	2	1	1
1	1	1	1
2	1	1	1
1	1	2	1
1	2	1	2
4	2	2	4
3	1	1	3
1	4	1	3
1	1	1	1
1	1	1	2
3	3	1	1
1	1	1	1
1	1	1	1
1	1	1	1

1	2	1	1
1	1	1	1
2	1	1	1
4	4	4	4
2	2	2	2
	3	3	2
1	1	1	1
2	2	2	2
1	2	1	2
1	1	1	2
2	2	1	3
1	2	1	3
1	1	1	1
2	2	2	2
1	1	1	2
4	3	2	2
3	2	2	2
3	1	3	4
2	2	1	2
4	4	4	4
2	1	1	1
2	2	1	2
4	4	2	4
2	3	1	1
3	2	2	2
2	3	1	2
1	2	1	4
1	1	1	1
1	1	1	1
1	3	2	1

1	1	1	1
2	2	1	3
2	3	3	4
1	1	1	1
1	1	1	1
1	1	1	1
1	1	1	2
2	1	1	1
1	1	1	1
3	2	1	4
2	2	1	1
2	3	1	1
3	2	2	2

mental health17	mental health18	mental health19	mental health20
1	1	1	1
1	1	1	1
1	1	1	1
3	2	1	1
3	2	4	1
1	2	1	1
4	4	1	4
4	4	1	4
4	4	1	4
1	1	1	1
1	2	1	1
1	2	1	2
1	2	1	1
1	3	3	2
1	2	3	1
2	3	2	2
1	4	4	4
1	1	1	1
2	4	2	3
1	1	1	1
1	2	1	2
1	1	1	1
1	3	1	1
4	4	1	1
1	1	1	1
1	3	1	1
3	3	2	2
2	2	2	2
1	3	1	1
1	2	3	1
1	2	1	1
1	1	1	1
3	1	4	1
1	2	1	1
1	1	1	1
1	3	4	1
2	1	2	2
1	1	1	1
3	3	1	1

3	4	1	4
2	2	2	2
4	4	1	1
1	1	4	1
1	4	4	1
1	1	1	1
2	3	1	1
1	1	1	1
1	1	1	1
1	1	1	1
1	2	4	1
2	1	1	1
1	2	1	1
4	3	1	2
2	3	1	2
1	2	1	1
4	2	1	1
	3	1	1
1	2	1	2
1	1	1	1
1	3	1	1
1	1	1	1
1	4	1	1
1	2	1	1
1	3	1	1
1	3	2	4
1	3	1	3
2	3	2	2
1	3	1	1
1	2	1	1
2	1	1	1
1	4	1	1
1	3	1	1
1	2	1	1
1	1	1	1
3	4	4	1
1	2	1	1
3	2	2	2
1	3	1	1
4	2	1	4
2	2	1	1

4	4	3	4
1	3	1	1
1	3	1	1
4	3	4	3

1	4	2	1
2	2	1	1
1	2	1	1
4	3	1	1
1	2	2	1
1	1	1	1
1	3	1	1
1	2	2	1
2	2	2	1
1	1	1	2
3	3	3	3
1	2	1	1
1	2	1	2

1	2	1	1
2	1	1	1
1	3	1	1
3	3	4	3
3	3	2	1
1	2	1	1
1	4	1	1
1	1	1	1
1	2	1	1
1	1	1	1
1	2	1	1
2	3	2	1
2	2	1	1
1	1	1	1
2	2	2	2
1	3	2	1
1	3	2	1
3	3	2	2
1	3	1	1
1	1	1	1
3	2	3	3
4	4	4	3
1	3	3	1
2	3	3	2
2	4	1	2

1	3	1	1
2	4	2	4
1	1	1	1
1	2	2	1
1	2	1	1
		1	1
1	3	1	3
1	4	4	4
2	1	1	1
1	4	2	2
1	1	1	1
1	1	1	1
1	1	1	1
1	4	1	1
2	1	1	1
1	1	1	1
2	3	2	1
1	3	1	1
2	2	2	2
1	1	1	1
1	1	1	1
3	3	3	3

1	1	1	1
1	2	1	2
1	1	1	1
1	3	1	1
4	4	1	1
1	1	1	1
1	3	1	1
3	3	2	2
2	2	2	2
1	3	1	1

1	2	3	1
1	2	1	1
1	1	1	1
3	1	4	1
1	2	1	1
1	1	1	1
1	3	4	1
2	1	2	2
1	1	1	1
3	3	1	1
3	4	1	4
2	2	2	2
4	4	1	1
1	1	4	1
1	4	4	1
1	1	1	1
2	3	1	1

1	1	1	1
1	1	1	1
1	1	1	1
1	2	4	1
2	1	1	1
1	2	1	1
4	3	1	2
2	3	1	2
1	2	1	1
4	2	1	1
	3	1	1
1	2	1	2
1	1	1	1
1	3	1	1
1	1	1	1

1	4	1	1
1	2	1	1
1	3	1	1
1	3	2	4
1	3	1	3
2	3	2	2
1	3	1	1
1	2	1	1
2	1	1	1
1	4	1	1
1	3	1	1
1	2	1	1
1	1	1	1
3	4	4	1
1	2	1	1
3	2	2	2
1	3	1	1
4	2	1	4
2	2	1	1
4	4	3	4
1	3	1	1
1	3	1	1
4	3	4	3
1	4	2	1
2	2	1	1
1	2	1	1
4	3	1	1
1	2	2	1
1	1	1	1
1	3	1	1

1	1	1	1
1	3	1	1
1	4	2	2
1	1	1	1
1	1	1	1
1	1	1	1
1	4	1	1
2	1	1	1
1	1	1	1
2	3	2	1
1	3	1	1
1	4	2	1
2	2	1	1

mental health21
1
1
1
3
4
1
4
4
3
1
2
2
1
4
1
1
1
1
2
1
2
1
1
4
1
1
2
2
1
1
1
1
1
1
1
1
2
1
3

3
2

1
1
1
1

1
1
3
1
1
1
4
3
1
1
1
1
1
1
1
2

1
1
4
3
2

1
2
1
1
1
1
4
2
3
2
2
1
1

4
2
2
4

1
1
1
3

1
1
1
2
1
4
1
1

2
1
1
3
3
2
1
1
1
1
1
1
1
1
2
1
1
2
1
1
2
4
1
2
3

1
3
1
2
1
1
1
2
1

1
1
1
1
1
2
1
1
1
1
1
1
3

1
2
1
1
4
1
1
2
2
1

1
1
1
1
1
1
1
2
1
3
3
2

1
1
1
1

1
1
3
1
1
1
4
3
1
1
1
1
1
1
1

2

1
1
4
3
2

1
2
1
1
1
1
4
2
3
2
2
1
1
4
2
2
4

1
1
1
3

1
1

1
1
1
1
1
1
1
2
1
1
1
1
1
