# Supplementary material for: Coping with identity threat and health literacy on the quality of life and mental health in students: Structural equation modeling
Source: Neuropsychopharmacol Rep. 2023 Mar 14;43(2):195–201. doi: 10.1002/npr2.12328 (PMC10275290; doi:10.1002/npr2.12328)
Supplement: Supplementary file 2 — Table S2 [file NPR2-43-195-s001.pdf]

Variable Information

Variable	Position	Label	Measurement Level	Role	Column Width	Alignment	Print Format	Write Format
AGE	1	<none>	Scale	Input	12	Right	F10	F10
COURSE	2	field	Nominal	Input	50	Left	A63	A63
TERM	3	term	Nominal	Input	41	Left	A41	A41
CHILD	4	<none>	Nominal	Input	12	Right	F10	F10
JOBFA	5	<none>	Nominal	Input	42	Left	A42	A42
residene	6	residence	Nominal	Input	30	Left	A30	A30
AQ1	7	quality of life	Nominal	Input	12	Right	F2	F2
AQ2	8	<none>	Nominal	Input	12	Right	F2	F2
AQ3	9	<none>	Nominal	Input	12	Right	F2	F2
AQ4	10	<none>	Nominal	Input	12	Right	F2	F2
AQ5	11	<none>	Nominal	Input	12	Right	F2	F2
AQ6	12	<none>	Nominal	Input	12	Right	F2	F2
AQ7	13	<none>	Nominal	Input	12	Right	F2	F2
AQ8	14	<none>	Nominal	Input	12	Right	F2	F2
AQ9	15	<none>	Nominal	Input	12	Right	F2	F2
AQ10	16	<none>	Nominal	Input	12	Right	F2	F2
AQ11	17	<none>	Nominal	Input	12	Right	F2	F2
AQ12	18	<none>	Nominal	Input	12	Right	F2	F2
AQ13	19	<none>	Nominal	Input	12	Right	F2	F2
AQ14	20	<none>	Nominal	Input	12	Right	F2	F2
AQ15	21	<none>	Nominal	Input	12	Right	F2	F2
AQ16	22	<none>	Nominal	Input	12	Right	F2	F2
AQ17	23	<none>	Nominal	Input	12	Right	F2	F2
AQ18	24	<none>	Nominal	Input	12	Right	F2	F2
AQ19	25	<none>	Nominal	Input	12	Right	F2	F2
AQ20	26	<none>	Nominal	Input	12	Right	F2	F2
AQ21	27	<none>	Nominal	Input	12	Right	F2	F2
AQ22	28	<none>	Nominal	Input	12	Right	F2	F2
AQ23	29	<none>	Nominal	Input	12	Right	F2	F2
AQ24	30	<none>	Nominal	Input	12	Right	F2	F2
AQ25	31	<none>	Nominal	Input	12	Right	F2	F2
AQ26	32	<none>	Nominal	Input	12	Right	F2	F2
BL1	33	Health litracy	Nominal	Input	12	Right	F2	F2
BL2	34	<none>	Nominal	Input	12	Right	F2	F2

BL3	35	<none>	Nominal	Input	12	Right	F2	F2
BL4	36	<none>	Nominal	Input	12	Right	F2	F2
BL5	37	<none>	Nominal	Input	12	Right	F2	F2
BL6	38	<none>	Nominal	Input	12	Right	F2	F2
BL7	39	<none>	Nominal	Input	12	Right	F2	F2
BL8	40	<none>	Nominal	Input	12	Right	F2	F2
BL9	41	<none>	Nominal	Input	12	Right	F2	F2
BL10	42	<none>	Nominal	Input	12	Right	F2	F2
BL11	43	<none>	Nominal	Input	12	Right	F2	F2
BL12	44	<none>	Nominal	Input	12	Right	F2	F2
BL13	45	<none>	Nominal	Input	12	Right	F2	F2
BL14	46	<none>	Nominal	Input	12	Right	F2	F2
BL15	47	<none>	Nominal	Input	12	Right	F2	F2
BL16	48	<none>	Nominal	Input	12	Right	F2	F2
BL17	49	<none>	Nominal	Input	12	Right	F2	F2
BL18	50	<none>	Nominal	Input	12	Right	F2	F2
BL19	51	<none>	Nominal	Input	12	Right	F2	F2
BL20	52	<none>	Nominal	Input	12	Right	F2	F2
BL21	53	<none>	Nominal	Input	12	Right	F2	F2
BL22	54	<none>	Nominal	Input	12	Right	F2	F2
BL23	55	<none>	Nominal	Input	12	Right	F2	F2
BL24	56	<none>	Nominal	Input	12	Right	F2	F2
BL25	57	<none>	Nominal	Input	12	Right	F2	F2
BL26	58	<none>	Nominal	Input	12	Right	F2	F2
BL27	59	<none>	Nominal	Input	12	Right	F2	F2
BL28	60	<none>	Nominal	Input	12	Right	F2	F2
BL29	61	<none>	Nominal	Input	12	Right	F2	F2
BL30	62	<none>	Nominal	Input	12	Right	F2	F2
BL31	63	<none>	Nominal	Input	12	Right	F2	F2
BL32	64	<none>	Nominal	Input	12	Right	F2	F2
BL33	65	<none>	Nominal	Input	12	Right	F2	F2
CC1	66	coping	Nominal	Input	12	Right	F2	F2
CC2	67	<none>	Nominal	Input	12	Right	F2	F2
CC3	68	<none>	Nominal	Input	12	Right	F2	F2
CC4	69	<none>	Nominal	Input	12	Right	F2	F2
CC5	70	<none>	Nominal	Input	12	Right	F2	F2
CC6	71	<none>	Nominal	Input	12	Right	F2	F2
CC7	72	<none>	Nominal	Input	12	Right	F2	F2

CC8	73	<none>	Nominal	Input	12	Right	F2	F2
CC9	74	<none>	Nominal	Input	12	Right	F2	F2
CC10	75	<none>	Nominal	Input	12	Right	F2	F2
CC11	76	<none>	Nominal	Input	12	Right	F2	F2
CC12	77	<none>	Nominal	Input	12	Right	F2	F2
CC13	78	<none>	Nominal	Input	12	Right	F2	F2
CC14	79	<none>	Nominal	Input	12	Right	F2	F2
CC15	80	<none>	Nominal	Input	12	Right	F2	F2
CC16	81	<none>	Nominal	Input	12	Right	F2	F2
CC17	82	<none>	Nominal	Input	12	Right	F2	F2
CC18	83	<none>	Nominal	Input	12	Right	F2	F2
CC19	84	<none>	Nominal	Input	12	Right	F2	F2
CC20	85	<none>	Nominal	Input	12	Right	F2	F2
CC21	86	<none>	Nominal	Input	12	Right	F2	F2
DD1	87	mental disorder	Nominal	Input	12	Right	F2	F2
DD2	88	<none>	Nominal	Input	12	Right	F2	F2
DD3	89	<none>	Nominal	Input	12	Right	F2	F2
DD4	90	<none>	Nominal	Input	12	Right	F2	F2
DD5	91	<none>	Nominal	Input	12	Right	F2	F2
DD6	92	<none>	Nominal	Input	12	Right	F2	F2
DD7	93	<none>	Nominal	Input	12	Right	F2	F2
DD8	94	<none>	Nominal	Input	12	Right	F2	F2
DD9	95	<none>	Nominal	Input	12	Right	F2	F2
DD10	96	<none>	Nominal	Input	12	Right	F2	F2
DD11	97	<none>	Nominal	Input	12	Right	F2	F2
DD12	98	<none>	Nominal	Input	12	Right	F2	F2
DD13	99	<none>	Nominal	Input	12	Right	F2	F2
DD14	100	<none>	Nominal	Input	12	Right	F2	F2
DD15	101	<none>	Nominal	Input	12	Right	F2	F2
DD16	102	<none>	Nominal	Input	12	Right	F2	F2
DD17	103	<none>	Nominal	Input	12	Right	F2	F2
DD18	104	<none>	Nominal	Input	12	Right	F2	F2
DD19	105	<none>	Nominal	Input	12	Right	F2	F2
DD20	106	<none>	Nominal	Input	12	Right	F2	F2
DD21	107	<none>	Nominal	Input	12	Right	F2	F2

Variables in the working file
